# The ongoing evolution of variants of concern and interest of SARS-CoV-2 in Brazil revealed by convergent indels in the amino (N)-terminal domain of the spike protein

**DOI:** 10.1093/ve/veab069

**Published:** 2021-08-14

**Authors:** Paola Cristina Resende, Felipe G Naveca, Roberto D Lins, Filipe Zimmer Dezordi, Matheus V. F Ferraz, Emerson G Moreira, Danilo F Coêlho, Fernando Couto Motta, Anna Carolina Dias Paixão, Luciana Appolinario, Renata Serrano Lopes, Ana Carolina da Fonseca Mendonça, Alice Sampaio Barreto da Rocha, Valdinete Nascimento, Victor Souza, George Silva, Fernanda Nascimento, Lidio Gonçalves Lima Neto, Fabiano Vieira da Silva, Irina Riediger, Maria do Carmo Debur, Anderson Brandao Leite, Tirza Mattos, Cristiano Fernandes da Costa, Felicidade Mota Pereira, Cliomar Alves dos Santos, Darcita Buerger Rovaris, Sandra Bianchini Fernandes, Adriano Abbud, Claudio Sacchi, Ricardo Khouri, André Felipe Leal Bernardes, Edson Delatorre, Tiago Gräf, Marilda Mendonça Siqueira, Gonzalo Bello, Gabriel L Wallau

**Affiliations:** Laboratory of Respiratory Viruses and Measles (LVRS), Instituto Oswaldo Cruz, FIOCRUZ-Rio de Janeiro, Av. Brasil, 4365 - Manguinhos, Rio de Janeiro 21040-900, Brazil; Laboratório de Ecologia de Doenças Transmissíveis na Amazônia (EDTA), Instituto Leônidas e Maria Deane, FIOCRUZ-Amazonas, Rua Teresina, 476. Adrianópolis, Manaus 69.057-070, Brazil; Department of Virology, Instituto Aggeu Magalhães, FIOCRUZ-Pernambuco, Av. Professor Moraes Rego, s/n – Cidade Universitária, Recife 50.740-465, Brazil; Departamento de Entomologia, Instituto Aggeu Magalhães, FIOCRUZ-Pernambuco, Av. Professor Moraes Rego, s/n – Cidade Universitária, Recife 50.740-465, Brazil; Núcleo de Bioinformática (NBI), Instituto Aggeu Magalhães FIOCRUZ-Pernambuco, Av. Professor Moraes Rego, s/n – Cidade Universitária, Recife 50.740-465, Brazil; Department of Virology, Instituto Aggeu Magalhães, FIOCRUZ-Pernambuco, Av. Professor Moraes Rego, s/n – Cidade Universitária, Recife 50.740-465, Brazil; Department of Fundamental Chemistry, Federal University of Pernambuco, Av. Professor Moraes Rego, s/n – Cidade Universitária, Recife 50.740-560, Brazil; Department of Virology, Instituto Aggeu Magalhães, FIOCRUZ-Pernambuco, Av. Professor Moraes Rego, s/n – Cidade Universitária, Recife 50.740-465, Brazil; Department of Fundamental Chemistry, Federal University of Pernambuco, Av. Professor Moraes Rego, s/n – Cidade Universitária, Recife 50.740-560, Brazil; Department of Virology, Instituto Aggeu Magalhães, FIOCRUZ-Pernambuco, Av. Professor Moraes Rego, s/n – Cidade Universitária, Recife 50.740-465, Brazil; Department of Fundamental Chemistry, Federal University of Pernambuco, Av. Professor Moraes Rego, s/n – Cidade Universitária, Recife 50.740-560, Brazil; Laboratory of Respiratory Viruses and Measles (LVRS), Instituto Oswaldo Cruz, FIOCRUZ-Rio de Janeiro, Av. Brasil, 4365 - Manguinhos, Rio de Janeiro 21040-900, Brazil; Laboratory of Respiratory Viruses and Measles (LVRS), Instituto Oswaldo Cruz, FIOCRUZ-Rio de Janeiro, Av. Brasil, 4365 - Manguinhos, Rio de Janeiro 21040-900, Brazil; Laboratory of Respiratory Viruses and Measles (LVRS), Instituto Oswaldo Cruz, FIOCRUZ-Rio de Janeiro, Av. Brasil, 4365 - Manguinhos, Rio de Janeiro 21040-900, Brazil; Laboratory of Respiratory Viruses and Measles (LVRS), Instituto Oswaldo Cruz, FIOCRUZ-Rio de Janeiro, Av. Brasil, 4365 - Manguinhos, Rio de Janeiro 21040-900, Brazil; Laboratory of Respiratory Viruses and Measles (LVRS), Instituto Oswaldo Cruz, FIOCRUZ-Rio de Janeiro, Av. Brasil, 4365 - Manguinhos, Rio de Janeiro 21040-900, Brazil; Laboratory of Respiratory Viruses and Measles (LVRS), Instituto Oswaldo Cruz, FIOCRUZ-Rio de Janeiro, Av. Brasil, 4365 - Manguinhos, Rio de Janeiro 21040-900, Brazil; Laboratório de Ecologia de Doenças Transmissíveis na Amazônia (EDTA), Instituto Leônidas e Maria Deane, FIOCRUZ-Amazonas, Rua Teresina, 476. Adrianópolis, Manaus 69.057-070, Brazil; Laboratório de Ecologia de Doenças Transmissíveis na Amazônia (EDTA), Instituto Leônidas e Maria Deane, FIOCRUZ-Amazonas, Rua Teresina, 476. Adrianópolis, Manaus 69.057-070, Brazil; Laboratório de Ecologia de Doenças Transmissíveis na Amazônia (EDTA), Instituto Leônidas e Maria Deane, FIOCRUZ-Amazonas, Rua Teresina, 476. Adrianópolis, Manaus 69.057-070, Brazil; Laboratório de Ecologia de Doenças Transmissíveis na Amazônia (EDTA), Instituto Leônidas e Maria Deane, FIOCRUZ-Amazonas, Rua Teresina, 476. Adrianópolis, Manaus 69.057-070, Brazil; Laboratório Central de Saúde Pública do Estado do Maranhão (LACEN-MA), Rua João Luís, Bairro Diamente, Sao Luis 65020-320, Brazil; Laboratório Central de Saúde Pública do Estado do Maranhão (LACEN-MA), Rua João Luís, Bairro Diamente, Sao Luis 65020-320, Brazil; Laboratório Central de Saúde Pública do Estado do Paraná (LACEN-PR), Rua Ubaldino do Amaral 545 - Alto da XV, Curitiba 80060-190, Brazil; Laboratório Central de Saúde Pública do Estado do Paraná (LACEN-PR), Rua Ubaldino do Amaral 545 - Alto da XV, Curitiba 80060-190, Brazil; Laboratório Central de Saúde Pública do Estado do Alagoas (LACEN-AL), Av. Marechal Castelo Branco, 1773 Jatiúca, Alagoas, 57030340 Brazil; Laboratório Central de Saúde Pública do Amazonas (LACEN-AM), Rua Emílio Moreira, 528 - Centro, Manaus 69020-040, Brazil; Fundação de Vigilância em Saúde do Amazonas, Av. Torquato Tapajós, 4.010 Colônia Santo Antônio, Manaus 69.093-018, Brazil; Laboratório Central de Saúde Pública do Estado da Bahia (LACEN-BA), Rua Waldemar Falcão, 123 - Bairro Brotas, Salvador 40295-001, Brazil; Laboratório Central de Saúde Pública do Estado de Sergipe (LACEN-SE), Rua Campo do Brito, 551 - Bairro São José, Aracajú, Sergipe 49020-380, Brazil; Laboratório Central de Saúde Pública do Estado de Santa Catarina (LACEN-SC), Avenida Rio Branco, 152 – Fundos, Florianópolis, Santa Catarina 88015-201, Brazil; Laboratório Central de Saúde Pública do Estado de Santa Catarina (LACEN-SC), Avenida Rio Branco, 152 – Fundos, Florianópolis, Santa Catarina 88015-201, Brazil; Instituto Adolfo Lutz, Av. Dr. Arnaldo, 351, São Paulo 01246-000, Brazil; Instituto Adolfo Lutz, Av. Dr. Arnaldo, 351, São Paulo 01246-000, Brazil; Laboratório de Enfermidades Infecciosas Transmitidas por Vetores, Instituto Gonçalo Moniz, FIOCRUZ-Bahia, Rua Waldemar Falcão, 121, Candeal, Salvador, Bahia 40296-710 , Brazil; Laboratório Central de Saúde Pública do Estado de Minas Gerais (LACEN-MG), Rua Conde Pereira Carneiro, 80 - Gameleira, Belo Horizonte 30510-010, Brazil; Departamento de Biologia, Centro de Ciências Exatas, Naturais e da Saúde, Universidade Federal do Espírito Santo, Av. Fernando Ferrari, 514 - Goiabeira, Alegre 29075-910, Brazil; Plataforma de Vigilância Molecular, Instituto Gonçalo Moniz, FIOCRUZ-Bahia, Rua Waldemar Falcão, 121, Candeal, Salvador 40296-710, Brazil; Laboratory of Respiratory Viruses and Measles (LVRS), Instituto Oswaldo Cruz, FIOCRUZ-Rio de Janeiro, Av. Brasil, 4365 - Manguinhos, Rio de Janeiro 21040-900, Brazil; Laboratório de AIDS e Imunologia Molecular, Instituto Oswaldo Cruz, FIOCRUZ-Rio de Janeiro, Av. Brasil, 4365 - Manguinhos, Rio de Janeiro 21040-900, Brazil; Departamento de Entomologia, Instituto Aggeu Magalhães, FIOCRUZ-Pernambuco, Av. Professor Moraes Rego, s/n – Cidade Universitária, Recife 50.740-465, Brazil; Núcleo de Bioinformática (NBI), Instituto Aggeu Magalhães FIOCRUZ-Pernambuco, Av. Professor Moraes Rego, s/n – Cidade Universitária, Recife 50.740-465, Brazil

**Keywords:** COVID-19, pandemics, antibody escape, SARS-CoV-2, community transmission

## Abstract

Mutations at both the receptor-binding domain (RBD) and the amino (N)-terminal domain (NTD) of the Severe Acute Respiratory Syndrome Coronavirus 2 (SARS-CoV-2) Spike (S) glycoprotein can alter its antigenicity and promote immune escape. We identified that SARS-CoV-2 lineages circulating in Brazil with mutations of concern in the RBD independently acquired convergent deletions and insertions in the NTD of the S protein, which altered the NTD antigenic-supersite and other predicted epitopes at this region. Importantly, we detected the community transmission of different P.1 lineages bearing NTD indels ∆69-70 (which can impact several SARS-CoV-2 diagnostic protocols), ∆144 and ins214ANRN, and a new VOI N.10 derived from the B.1.1.33 lineage carrying three NTD deletions (∆141–144, ∆211, and ∆256–258). These findings support that the ongoing widespread transmission of SARS-CoV-2 in Brazil generates new viral lineages that might be more resistant to antibody neutralization than parental variants of concern.

## 1. Introduction

Recurrent deletions in the amino (N)-terminal domain (NTD) of the spike (S) glycoprotein of SARS-CoV-2 have been identified during long-term infection of immunocompromised patients (Avanzato et al. 2020; Choi et al. 2020; Kemp et al. 2021; McCarthy et al. 2021) as well as during extended human-to-human transmission (McCarthy et al. 2021). Most of those deletions (90 per cent) maintain the reading frame and cover four recurrent deletion regions (RDRs) within the NTD at positions 60–75 (RDR1), 139–146 (RDR2), 210–212 (RDR3), and 242–248 (RDR4) of the S protein (McCarthy et al. 2021). The RDRs that occupy defined antibody epitopes within the NTD and RDR regions might alter antigenicity (McCarthy et al. 2021). Interestingly, the RDRs overlap with four NTD Indel Regions (`, Tem um ' e um espaco a mais IR-2 to IR-5) that are prone to gain or lose short nucleotide sequences during sarbecoviruses evolution known to infect animals and humans (Garry et al. 2021; Holmes et al. 2021).

Since late 2020, several more transmissible variants of concern (VOCs) and also variants of interest (VOIs) with convergent mutations at the receptor-binding domain (RBD) of the S protein (particularly E484K and N501Y) arose independently in humans (Rambaut et al. 2020; Tegally et al. 2021). Some VOCs also displayed NTD deletions such as lineages B.1.1.7 (RDR2 ∆144), B.1.351 (RDR4 ∆242-244), and P.3 (RDR2 ∆141-143) that were initially detected in the UK, South Africa, and the Philippines, respectively (McCarthy et al. 2021). The VOCs B.1.1.7 and B.1.351 are resistant to neutralization by several anti-NTD monoclonal antibodies (mAbs), and NTD deletions at RDR2 and RDR4 are essential for such phenotype (Collier et al. 2021; Gobeil et al. 2021; McCallum et al. 2021: 2; Wang et al. 2021a: 7, 2021b; 2021). Thus, NTD mutations and deletions represent an important mechanism of immune evasion and accelerate SARS-CoV-2 adaptive evolution in humans.

Several SARS-CoV-2 variants with mutations in the RBD have been described in Brazil, including the VOC P.1 (now also known as gamma) (Faria et al. 2021) and the VOIs P.2 (Voloch et al. 2021) and N.9 (Resende et al. 2021), but none of them displayed indels in the NTD. Importantly, although the VOC P.1 displayed NTD mutations (L18F) that abrogate binding of some anti-NTD mAbs (McCallum et al. 2021: 2) and further showed reduced binding to RBD-directed antibodies, it is more susceptible to anti-NTD mAbs than other VOCs (Collier et al. 2021; Dejnirattisai et al. 2021: 2; Gobeil et al. 2021; McCallum et al. 2021: 2; Wang et al. 2021a: 7, 2021b; 2021). In this study, we characterized the emergence of RDR variants within the VOC P.1, the VOI P.2, and a new VOI descendant of lineage B.1.1.33 (designated as N.10) that were circulating in Brazil between November 2020 and February 2021.

## 2. Results

### 2.1 Emergence of SARS-CoV-2 VOC and VOI with NTD indels in Brazil

The Fiocruz COVID-19 Genomic Surveillance Network identified forty-three SARS-CoV-2 sequences from seven different Brazilian states that harbor a variable combination of mutations in the RBD (K417T, E484K, and N501Y) and indels in the NTD region of the S protein and were classified within lineages P.1 (*n* = 21), N.10 (*n* = 17), and P.2 (*n* = 2) (Supplementary Appendix Table A1). All NTD indels here detected were well-supported by several high-quality reads longer than the indels (Supplementary Appendix Table A1 and Supplementary Fig. S1), indicating that such genomic variations are real and not sequencing artifacts. The most frequent deletions in lineage P.1 were ∆69-70 in the RDR1; ∆144 and ∆141-144 in the RDR2; ∆189-190 and ∆242-244 in the RDR4. We also detected four P.1 genomes bearing an ins214ANRN insertion upstream to RDR3 that were part of a complex family of P.1-related variants that share several, but not all, lineage-defining mutations of VOC P.1 (Naveca et al. 2021). The two NTD deletions in the VOI P.2 were ∆144 and ∆141-144. Inspection of sequences available at EpiCoV database in the GISAID (https://www.gisaid.org/) on 31 May 2021 retrieved a large number of lineage P.1 (*n* = 101) sequences and a more reduced number of B.1.1.28 (*n* = 18) or P.2 (*n* = 13) variants with similar NTD indels sampled in Brazil (Franceschi et al. 2021; Lamarca et al. 2021; Martins et al. 2021; Siqueira et al. 2021; Slavov et al. 2021; Tosta et al. 2021) (Table 1) and other countries (Supplementary Appendix Table A2).

**Table 1.** SARS-CoV-2 Brazilian variants with indels at NTD of the spike protein.

Lineage	RBD	NTD indel	*n*	Earliest sequence	Last sequence
B.1.1.28 and B.1.1.33	−	∆69-70	4	28 March 2021	29 March 2021
		∆144	7	19 June 2020	16 January 2021
		∆143-144	1	28 September 2020	−
		∆141-144	1	2 January 2021	−
P.2	E484K	∆144	2	14 January 2021	21 January 2021
		∆141-144	1	30 March 2021	−
P.1	K417T	∆69-70	16	27 January 2021	27 April 2021
	E484K	∆144	11	12 January 2021	12 April 2021
	N501Y	∆143-144	2	19 April 2021	26 April 2021
		∆141-144	3	26 January 2021	14 April 2021
		∆189-190	2	27 January 2021	15 March 2021
		∆242-244	1	23 February 2021	−
P.1 (P.1-like-I)	K417T E484K N501Y	ins214ANRN	6	23 December 2020	5 April 2021
N.10	V445A E484K	∆141-144 ∆211 ∆256-258	16	29 December 2020	26 April 2021

Sequencing depth plots of the samples bearing indels are available in Supplementary Fig. S1.

The emergence of NTD indels within lineage B.1.1.33 was rare. Our genomic survey identified 17 B.1.1.33 sequences that displayed both RBD mutations and NTD deletions and that were designated as a new PANGO lineage N.10 (Table 1). This new VOI N.10 exhibited fourteen lineage-defining genetic changes, including ten non-synonymous mutations, three in-frame deletions, and one frame-shifting 4nt deletion (Supplementary Appendix Table A3). Eight lineage-defining genetic changes were located in the S protein where two are non-synonymous mutations at the RBD (E484K and V445A), two non-synonymous mutations at the NTD (I210V and L212I), and deletions at RDR2 (∆141-144), RDR3 (∆211), and close to RDR4 (∆256-258) in the NTD. All N.10 sequences identified in our study were sampled in the Northeastern Brazilian state of Maranhão between 4 January and 1 May 2021. Inspection of sequences available at EpiCoV database retrieved seven additional N.10 sequences from the states of Maranhão (*n* = 3), Amapá (*n* = 2), and São Paulo (*n* = 2) sampled between 29 December 2020 and 26 April 2021 as well as only three additional B.1.1.33 Brazilian sequences carrying NTD-indels ∆141-144, ∆144, and ∆144-145 sampled between 8 March and 11 May 2021.

**Figure 1.**
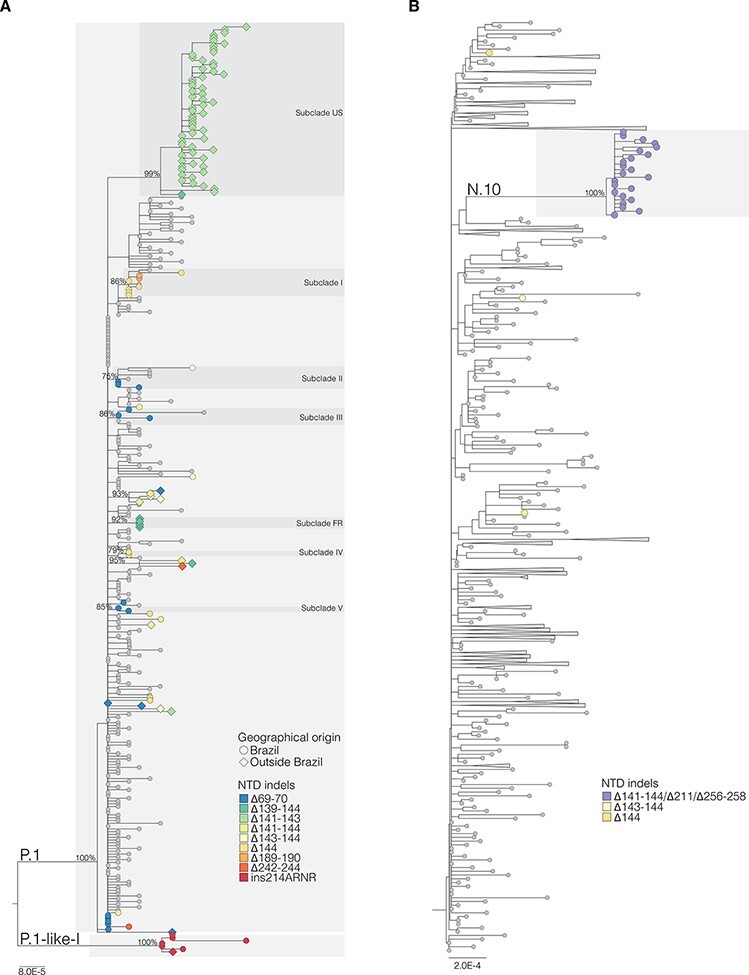
ML phylogenetic tree of whole-genome lineages P.1 (A) and B.1.1.33 (B) sequences from Brazil and other countries showing the recurrent emergence of indels at the NTD of the S protein. The color of the tips represents the type of NTD indels found in SARS-CoV-2 sequences from Brazil (circles) and in other countries (diamonds) as indicated in the legend at bottom right. The branch lengths are drawn to scale with the bars at the bottom indicating nucleotide substitutions per site. For visual clarity, some clades were collapsed into triangles. US—United States of America; FR—France.

The Maximum Likelihood (ML) phylogenetic analysis of lineage P.1 supports the recurrent emergence of most NTD variants, except for ∆189-190 and ins214ANRN (Fig. 1A). Among Brazilian P.1 variants with NTD deletions, 41 per cent appeared as singletons intermixed among non-deleted P.1 sequences, 47 per cent branched in three sub-clades (I–III) that also include non-deleted P.1 sequences, and 12 per cent branched in two subclades (IV and V) that only comprises sequences with NTD deletions. Sub-clade I (aLRT = 86 per cent) comprises ten sequences: six P.1+∆144 from Amazonas and Bahia states, two P.1+∆189-190, and two P.1. Sub-clade II (aLRT = 75 per cent) comprises eight sequences: three P.1+∆69-70 from São Paulo state plus one P.1+∆143-144 from Rio de Janeiro state and four P.1. Sub-clade III (aLRT = 86 per cent) comprises six sequences from São Paulo state: four P.1+∆69-70 plus two P.1. Sub-clade IV (aLRT = 79 per cent) comprises two P.1+∆144 sequences from São Paulo state, and sub-clade V (aLRT = 85 per cent) comprises two P.1+∆69-70 sequences from Santa Catarina state. This analysis also identified multiple clusters of P.1 variants with NTD deletions outside Brazil, including a large P.1+∆141-143 sub-clade in the USA (*n* = 61, aLRT = 99 per cent) and a small P.1+∆139-144 sub-clade in France (*n* = 4, aLRT = 92 per cent). The ML phylogenetic analysis of lineage B.1.1.33 confirms that all sequences belonging to VOI N.10 branched in highly supported (aLRT = 100 per cent) monophyletic clade (Fig. 1B).

### 2.2 Worldwide prevalence of SARS-CoV-2 variants with NTD indels

**Figure 2.**
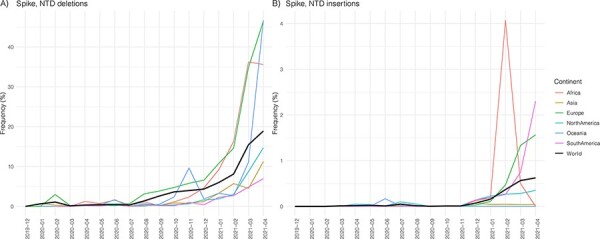
Temporal trend of the frequency of SARS-CoV-2 variants with NTD deletions (A) and insertions (B) circulating globally available at EpiCoV database in the GISAID (https://www.gisaid.org/) up to 31 May and sampled up to 30 April. Lineages B.1.1.7, B.1.351, and B.1.351-derived lineages were excluded from the analysis.

Inspection of sequences available at EpiCoV database in the GISAID (https://www.gisaid.org/) up to 31 May 2021 revealed that frequency of SARS-CoV-2 genomes with NTD deletions ∆69-70, ∆144, ∆141-144, ∆189-190, ∆242-244, and ∆256-258 emerged in many different lineages (Supplementary Appendix Table A4) and displayed a consistent increase over time worldwide, particularly from July 2020, even after removing genomes of VOCs B.1.1.7 and B.1.351 that were mostly associated with the widespread dissemination of NTD deletions ∆69-70/∆144 and ∆242-244, respectively (Fig. 2A). NTD insertions were much less prevalent than deletions, but their frequency also increased in 2021 (Fig. 2B). The ins214ANRN motif was exclusive of lineage P.1, but other ins214 motifs of three to four amino acids were detected in several lineages, including the VOC B.1.1.7 (Supplementary Appendix Table A5). Most ins214 motifs were unique, except for the ins214AAG and ins214TDR that arose independently in several lineages, including the A.2.5 (ins214AAG) and B.1.214.2 (ins214TDR) that spread in Central/North America and Europe, respectively.

### 2.3 NTD indels in SARS-CoV-2 and SARS-CoV-2-related coronavirus

**Figure 3.**
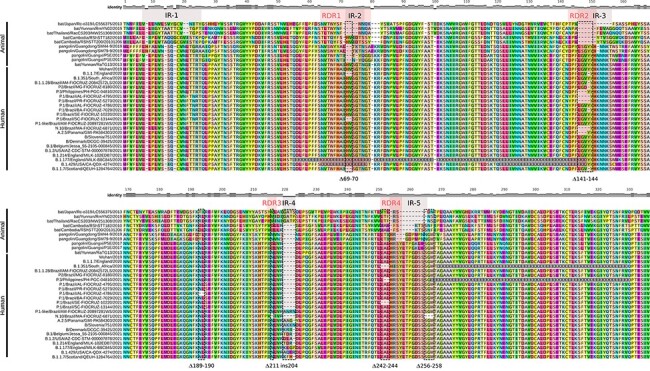
Amino acid alignment of Sarbecovirus NTD spike region up to amino acid 335 including some representative sequences of SARS-CoV-2 lineages harboring indels in the NTD and SARS-CoV-2-related coronavirus (SC2r-CoV) from bats and pangolins. IRs and RDRs positions (gray and red shaded areas, respectively) are approximations due to the high genetic variability in these alignment positions. Dotted rectangles highlight the indels identified in this study. The relative identity level estimated for each position of the alignment is displayed at the top.

To better understand the evolutionary context of NTD indels, we aligned the S protein of representative sequences of SARS-CoV-2 lineages with NTD indels and SARS-CoV-2-related coronavirus (SC2r-CoV) lineages from bats and pangolins (Wacharapluesadee et al. 2021). Inspection of the alignment confirms that most NTD indels detected in the SARS-CoV-2 lineages occur within IR previously defined in sarbecovirus (Fig. 3). The ∆141-144 occurs in the IR-3 located in the central part of the NTD, where some bat SC2r-CoVs also have deletions. The ∆211 and ins214 occur near the IR-4 where some bat SC2r-CoVs from China (RmYN02, ins214GATP), Thailand (RacCS203, ins214GATP), and Japan (Rc-o319, ins214GATS) displayed a four-amino-acid insertion. Although amino acid motifs at ins214 are very different across SARS-CoV-2 and SC2r-CoV lineages, the insertion size (three to four amino acids) was conserved. Deletions ∆242-244 and ∆256-258 occur immediately upstream and downstream to IR-5, respectively, where some bat and pangolin SC2r-CoV lineages also displayed deletions. Thus, NTD regions prone to gain indels during viral transmission among animals are the same as those detected during transmissions in humans.

### 2.4 NTD indels in SARS-CoV-2 and antibody binding

**Figure 4.**
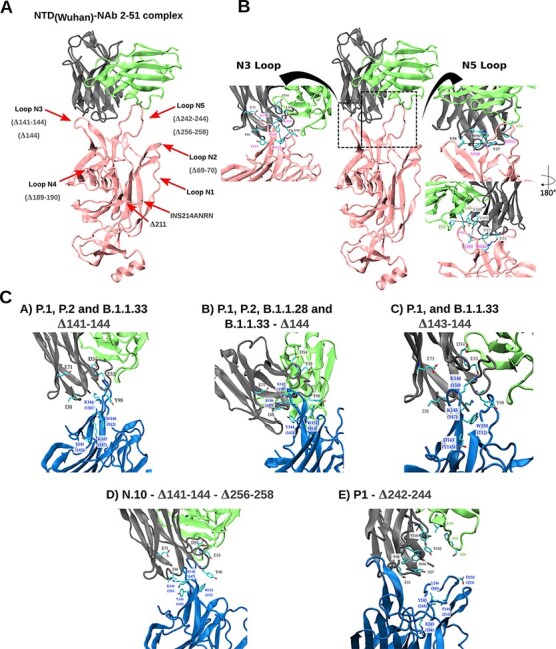
Representation of the spike NTD three-dimensional (3D) structure of wild-type (pink) and NTD-deleted variants (colored in blue) complexed to the NAb 2-51 heavy (gray) and light (green) chains. (A) Relative position of the five NTD loops (red arrows) and the NTD deletions detected in our sample. (B) Native interactions of mAb NAb 2-51 with N3 (left close-up) and N5 (right close-up) loops on the 3D structure of the wild-type spike NTD antigenic supersite. The N5 loop representation is also rotated 180° around its z-axis. (C) Potential interactions of mAb NAb 2-51 with N3 and N5 loops on the 3D structure of the spike NTD of N3- and N5-deleted variants. Residues making contact in the interface are depicted in the licorice representation, with carbon atoms in cyan, nitrogen atoms in blue, and oxygen atoms in red. The dotted lines indicate the interacting residue pairs.

Epitope mapping showed that neutralizing antibodies are primarily directed against the RBD and NTD of the S protein (Barnes et al. 2020; Liu et al. 2020; Piccoli et al. 2020; Voss et al. 2020; Wang et al. 2021). Some of the RBD mutations (K417T and E484K) detected in the VOCs and VOIs circulating in Brazil have been associated with increased resistance to neutralization by mAbs or polyclonal sera from convalescent and vaccinated subjects (Baum et al. 2020; Ferraz et al. 2021; Greaney et al. 2021; Hoffmann et al. 2021; Nelson et al. 2021). The RDR2 and RDR4 are located in the N3 (residues 141–156) and N5 (residues 246–260) loops that compose the NTD antigenic-supersite (Cerutti et al. 2021; Chi et al. 2020: 2). Deletions at those RDRs are also an essential mechanism for SARS-CoV-2-immune evasion of anti-NTD Abs (McCallum et al. 2021; McCarthy et al. 2021; Wang et al. 2021a: 7; 2021; Wibmer et al. 2021). To further visualize the potential impact of NTD deletions on immune recognition, we performed a modeling analysis of the binding interface between the NTD region and the NTD-directed neutralizing antibody (NAb) 2–51 derived from a convalescent donor (Liu et al. 2020; Cerutti et al. 2021). The NAb 2–51 interacts with the wild-type NTD antigenic-supersite (EPI_ISL_402124) through several contacts with loops N3 and N5, with a predominance of hydrophobic contacts and dispersion interactions in N5 and saline interactions in N3 (Fig. 4A and B).

Our analyses corroborate that deletions at RDR2/IR-3 (∆144, ∆143-144, and ∆141-144) and RDR4/IR-5 (∆242-244 and ∆256-258) detected in Brazilian sequences impact the N3 and N5 loops’ size and conformation, disrupting the native contacts and reducing the interacting hydrophobic surface accessible area, mainly due to the loss of the hydrophobic pocket (Fig. 4C). Indels around the N3/N5 loops resulted in a significant loss of interactions (both electrostatic and hydrophobic) that can dramatically impact the binding free energy and, therefore, the binding affinity between those NTD deletion variants and the NAb 2-51. Variant N.10 displayed the largest loss of interactions, followed by variants with deletions ∆242-244, ∆141-144, and ∆143-144/∆144 (Table 2). Loss of interactions for P.1/P.2 variants was larger than for B.1.1.28/33 variants with the same NTD deletions. The NTD indels ∆69-70, ∆189-190, ∆211, and ins214ANRN did not affect the NTD antigenic supersite (Fig. 4A). Still, they occur at other loops that comprise putative epitope regions covering residues 64–83, 168/173–188, and 209–216 (Supplementary Appendix Table A6) and lead to conformational changes (Supplementary Fig. S2), which might affect Ab binding outside the NTD antigenic supersite.

**Table 2.** Impact of indels on the binding between SARS-CoV-2 NTDs and NAb 2-51, expressed as loss of putative interactions.

Variant	Indel	ΔH-bond	ΔSalt-Bridge	Δπ-Stacking	ΔHydrophobic SASA [Å^2^]	Native contacts lost
B.1.1.28	∆144	−2	−3	−1	−103	Y145-Y98(H) K147-E71(H) K150-E53(H) K150-D54(H)
B.1.1.33					−108	
P.2					−104	
P.1					−111	
B.1.1.33	∆143-144	−1	−1	−1	−120	Y144-I30(H) Y145-98Y(H) K147-E71(H K150-D54(H)
P.1					−130	
B.1.1.33	∆141-144	−2	−3	−2	−305	Y144-I30(H) Y145-98Y(H) K147-E71(H) K150-E53(H) K150-D54(H) W152-98Y(H)
P.2					−317	
P.1					−313	
P.1	∆242-244	−3	−1	−2	−347	R246-E31(H) R246-Y27(H) Y248-Y27(H) Y248-W96(H) Y248-Y98(H) L249-Y27(H) P251-L46(L) P251-P55(L) P251-Y100(H) P251-Y102(H)
N.10	∆141-144 ∆211 ∆256-258	−3	−3	−2	−439	Y144-I30(H) Y145-Y98(H) K147-E71(H) K150-E53(H) K150-D54(H) W152-Y98(H) L249-Y27(H) P251-L46(L) P251-P55(L) P251-Y100(H) P251-Y102(H) D253-S56(L)

## 3. Discussion

Our study revealed that NTD deletions characteristic of VOCs B.1.1.7 (∆69-70 and ∆144) and B.1.351 (∆242-244) as well as other NTD indels (∆143-144, ∆141-144, ∆189-190, and ins214ANRN) occurred at multiple times during the evolution of lineage P.1 and also sporadically in lineages B.1.1.28, B.1.1.33, and P.2 in Brazil and worldwide. Most P.1 variants with similar NTD deletions detected in different countries were not closely related to each other, supporting that they arose independently. We detected a low level of community transmission of P.1+∆144 and P.1 + ins214 variants in the Amazonas state and of a P.1+∆69-70 variant in the Santa Catarina and São Paulo states. We also found evidence of local spread of a P.1+∆141-143 variant in the USA and a P.1+∆139-144 variant in France. Our study also revealed that a new lineage N.10 carrying multiple mutations with phenotypic implications in the RBD (E484K and V445A) and NTD (I210V, ∆211, L212I, ∆141-144, and ∆256-258) of the S protein evolved within lineage B.1.1.33 and constituted an emergent VOI that seems to be mostly restricted to the Northeastern Brazilian state of Maranhão.

The two most frequent NTD indels in lineage P.1 samples were ∆69-70 and ∆144, as observed in the VOC B.1.1.7. We detected the recurrent emergence of deletion ∆69-70 in Brazil (states of Santa Catarina and São Paulo), Aruba, Austria, Spain, and the USA and of deletion ∆144 in Brazil (states of Amazonas, Bahia, Rio de Janeiro, and São Paulo), Spain, and the USA. While NTD deletions ∆69-70 and ∆144 arose multiple times during the evolution of VOC P.1, the simultaneous presence of both mutations was only detected in one P.1 sequence from Spain. The detection of P.1 genomes with convergent NTD deletions with VOCs B.1.1.7 and B.1.351 in different countries from the Americas (Brazil, Aruba, and the USA) and Europe (Austria, France, and Spain) bring caution about the specificity of published or commercial real-time reverse transcription polymerase chain reaction (PCR) protocols to distinguish different VOCs. Indeed, we alert against using the failure to detect the S gene (due to mutation ∆69-70) by certain PCR tests, known as S gene target dropout (Bal et al. 2021; Korukluoglu et al. 2021), as a definitive proof of circulation of the VOC B.1.1.7 in Brazil or elsewhere.

We observed that SARS-CoV-2 variants harboring NTD indels have arisen in different lineage backgrounds, and its frequency has increased globally since mid-2020. Recent genomic findings showed a sudden landscape change in SARS-CoV-2 evolution since October 2020, coinciding with the independent emergence of VOCs carrying multiple convergent amino acid replacements at the RBD of the S protein (Martin et al. 2021). One hypothesis is that such a major selection pressure shift on the virus genome is driven by the increasing worldwide human population immunity acquired from natural SARS-CoV-2 infection that might also select for convergent deletions at NTD. Our findings suggest that P.1, P.2, and N.10 variants with NTD indels here detected might have evolved to escape from NAb against NTD and could be more resistant to neutralization than the parental viruses. Notably, the sequential acquisition of RBD and NTD mutations observed in the VOC P.1 recapitulates the evolution pattern of the VOC B.1.351 that first acquired RBD mutations E484K and N501Y and sometime later the NTD deletion ∆242-244 (Tegally et al. 2021).

Our *in silico* analyses suggest that several NTD indels detected at RDR2 and RDR4 probably abrogate the binding of NAb directed against the antigenic supersite and thus represent an adaptive mechanism of immune escape. Several studies of SARS-CoV-2 evolution *in vitro* and *ex vivo* also support this hypothesis. *In vitro* co-incubation of SARS-CoV-2 with highly neutralizing plasma from a coronavirus-19 (COVID-19) convalescent patient revealed an incremental resistance to neutralization followed by the stepwise acquisition of indels at N3/N5 loops (Andreano et al. 2020). SARS-CoV-2 challenge in hamsters previously treated with anti-NTD mAbs revealed the selection of two escape mutants harboring NTD deletions ∆143-144 and ∆141-144 (McCallum et al. 2021). Studies of intra-host SARS-CoV-2 evolution showed the emergence of viral variants with NTD deletions at RDR1 (∆69-70), RDR2 (∆144 and ∆141-144), and RDR4 (∆243-244) following the therapy of immuno-compromised hosts with convalescent plasma (Avanzato et al. 2020; Chen et al. 2021; Kemp et al. 2021; Khatamzas et al. 2021; McCarthy et al. 2021) and the emergence of similar NTD deletions (Δ141-143, Δ141-144, Δ145, and Δ211-212) during persistent SARS-CoV-2 infection in two individuals with partial humoral immunity (Truong et al. 2021). Finally, a recent study revealed the emergence of virus haplotypes bearing NTD deletions ∆144 and ∆141-144 following the development of autologous anti-NTD-specific antibodies during acute infection in one immunocompetent individual (Ko et al. 2021: 1).

The impact of indels at RDR1 and RDR3 on immune escape remains unknown as they did not affect the NTD antigenic supersite. Our *in silico* analyses supports that the NTD indels at RDR1 and RDR3 occur at external loops that comprise putative epitope regions and leads to conformational changes that might affect Ab binding outside the NTD antigenic supersite. A recent study found that some Ab from convalescent subjects directed against the NTD induce the open conformation of RBD and enhance the binding capacity of the S protein to ACE2 receptor and infectivity of SARS-CoV-2 (Liu et al. 2021). Notably, all the infectivity-enhancing Abs recognized a specific site covering residues 64–66, 187, and 213–214 of the NTD. Another study supports that the NTD deletion Δ69-70 is not an antibody evasion mechanism, but it increases the viral infectivity associated with enhanced incorporation of cleaved S into virions (Meng et al. 2021). Thus, indels around the RDR1 (Δ69-70) and RDR3 (Δ211 and ins214) might enhance the infectivity of SARS-CoV-2 lineages carrying RBD immune escape mutations.

Analyses of sarbecoviruses in bats and other species identified that the NTD RDRs of the SARS-CoV-2 overlap with four NTD IRs that are prone to gain or lose short nucleotide sequences during sarbecoviruses evolution (Holmes et al. 2021). Most NTD indels detected in the VOC P.1 and VOIs P.2/N.10 occurred in or adjacent to the sarbecoviruses IR-2 (∆69-70), IR-3 (∆144, ∆141-144), IR-4 (∆211 and ins214), and IR-5 (∆242-244 and ∆256-258). While NTD deletions in the IR-2, IR-3, and IR-5 were already detected in VOCs B.1.1.7, B.1.351, and B.1.617.2, the presence of indels in the IR-4 seems to be rare. The three- to four-amino-acid insertion noticed in the sub-clade P.1 + ins214ARNR and in other emergent SARS-CoV-2 lineages resembles the insertions observed in some bat SC2r-CoVs from China (ins214GATP), Thailand (ins214GATP), and Japan (ins214GATS). This not only supports that all IR remains evolutionarily active in SARS-CoV-2 but also demonstrates that insertions at IR, as the one observed at the furin cleavage site, are part of the natural evolutionary process of SARS-CoV-2.

In summary, our findings suggest that the SARS-CoV-2 VOCs and VOIs are continuously adapting and evolving in Brazil through the acquisition of Spike NTD indels. Some variants like P.1+∆69-70, P.1+∆144, P.1 + ins214ANRN, and N.10 might represent newly emergent VOC/VOI, and its community dissemination requires careful monitoring. Our findings also highlight the urgent need to address the SARS-CoV-2 vaccines’ efficacy toward emergent SARS-CoV-2 variants carrying RBD and NTD mutations and deletions of concern. Furthermore, the uncontrolled community transmission of SARS-CoV-2 in Brazil and other countries leads to the risk of the emergence of more transmissible variants. The recurrent rise of NTD ins214 variants in different SARS-CoV-2 lineages circulating in the Americas and Europe since November 2020 and its impact on vaccine efficacy also deserve further attention.

## 4. Material and methods

### 4.1 SARS-CoV-2 and SARS-CoV-2-related coronavirus (SC2r-CoV) sequences

Our genomic survey of SARS-CoV-2-positive samples sequenced by the Fiocruz COVID-19 Genomic Surveillance Network between 12 March 2020 and 28 June 2021 identified 21 sequences with mutations of concern in the RBD and indels in the NTD (Supplementary Appendix Table A1). The SARS-CoV-2 genomes were recovered using Illumina sequencing protocols as previously described (Nascimento et al. 2020; Resende et al. 2020). The FASTQ reads obtained were imported into the CLC Genomics Workbench version 20.0.4 (Qiagen A/S, Denmark), trimmed and mapped against the reference sequence EPI_ISL_402124 (hCoV-19/Wuhan/WIV04/2019) available in EpiCoV database in the GISAID (https://www.gisaid.org/). The alignment was refined using the InDels and Structural Variants module. Additionally, the same reads were imported in a different pipeline (Paiva et al. 2020) based on Bowtie2 and bcftools (Li 2011) mapping and consensus generation allowing us to further confirm the indels supported by paired-end reads, removing putative indels with less than 10× of sequencing depth and with mapping read quality score below 10 for all samples sequenced in this study. BAM files were used to generate sequencing coverage plots onto indels using the Karyoploter R package (Gel and Serra 2017). Sequences were combined with SARS-CoV-2 and SC2r-CoV from bats and pangolins available in the EpiCoV database in GISAID by 1 March 2021 (Supplementary Appendix Table A7). This study was approved by the FIOCRUZ-IOC (68118417.6.0000.5248 and CAAE 32333120.4.0000.5190), the Amazonas State University Ethics Committee (CAAE: 25430719.6.0000.5016), and the Brazilian Ministry of the Environment (MMA) A1767C3.

### 4.2 ML phylogenetic analyses

SARS-COV-2 P.1 and N.10 sequences here obtained were aligned with high quality (<1 per cent of N) and complete (>29 kb) lineages P.1, N.10, and B.1.1.33 Brazilian sequences that were available in EpiCoV database in the GISAID (https://www.gisaid.org/) on 31 May 2021. All P.1 sequences sampled worldwide available in the EpiCoV database that harbor the same NTD indels described in Brazilian sequences were also downloaded. Sequences were aligned using Clustal W (Larkin et al. 2007) and then subjected to ML phylogenetic analysis using IQ-TREE v2.1.2 (Nguyen et al. 2015) under the general time-reversible model of nucleotide substitution with a gamma-distributed rate variation among sites, four rate categories (G4), a proportion of invariable sites (I) and empirical base frequencies (F) nucleotide substitution model, as selected by the ModelFinder application (Kalyaanamoorthy et al. 2017). The branch support was assessed by the approximate likelihood-ratio test based on the Shimodaira–Hasegawa-like procedure with 1,000 replicates. Indels were not treated as informative characters for phylogenetic reconstructions. The S amino acid sequences from selected SARS-CoV-2 and SC2r-CoV lineages available in the EpiCoV database were also aligned using Clustal W (Larkin et al. 2007) and adjusted by visual inspection.

### 4.3 NTD indels screening in the world dataset

The EpiCov worldwide metadata package was downloaded from GISAID (https://www.gisaid.org/) on 31 May. Along with several patient metadata, the file included all amino acid substitutions (including indels) relative to hCoV-19/Wuhan/WIV04/2019 genome reference. Entries without complete sampling date and from non-human hosts were excluded. Sequences were then filtered for the presence of insertions or deletions at positions 13–305 of the spike protein. Aiming to show the continuous emergence of NTD indels, lineages B.1.1.7, B.1.351, and B.1.351-derived lineages (sublineages) were excluded from the analysis, since they are known to harbor indels in the NTD and they represent a large amount of the EpiCov database. Due to the reduced number of samples from May in comparison with previous months, the proportion of sequences carrying NTD indels was only plotted up to 30 April. Data cleaning and processing were performed in R and plotted using the ggplot2 package.

### 4.4 Structural modeling

The resolved crystallographic structure of SARS-CoV-2 NTD protein bound to the NAb 2-51 was retrieved from the Protein Databank (PDB) under the accession code 7L2C (Cerutti et al. 2021). Missing residues of the chain A, corresponding to the NTD coordinates, were modeled using the user template mode of the Swiss-Model webserver (https://swissmodel.expasy.org/) (Waterhouse et al. 2018) and was used as starting structure for the NTD wild-type. This structure was then used as a template to model the NTD variants using the Swiss-Model webserver. The modeled structures of the NTDs variants were superimposed onto the coordinates of the PDB ID 7L2C to visualize the differences between the NTD-antibody binding interfaces. Image rendering was carried out using Visual Molecular Dynamics software (Humphrey, Dalke, and Schulten 1996). The NTD–antibody complexes were geometry optimized using a maximum of 5,000 steps or until it reached a convergence value of 0.001 REU (Rosetta energy units) using the limited-memory BroydenFletcher–Goldfarb–Shanno algorithm, complying with the Armijo–Goldstein condition, as implemented in the Rosetta suite of software 3.12 (Leaver-Fay et al. 2011). Geometry optimization was accomplished through the atomistic Rosetta energy function 2015 (REF15), while preserving backbone torsion angles. Protein–protein interface analyses were performed using the Protein Interactions Calculator webserver (http://pic.mbu.iisc.ernet.in/) (Tina, Bhadra, and Srinivasan 2007), the ‘Protein interfaces, surfaces and assemblies’ service at the European Bioinformatics Institute (https://www.ebi.ac.uk/pdbe/pisa/pistart.html) (Krissinel and Henrick 2007), and the InterfaceAnalyzer protocol of the Rosetta package interfaced with the RosettaScripts scripting language (Fleishman et al. 2011). For the interfaceAnalyzer, the maximum solvent accessible surface area (SASA) that is allowed for an atom to be defined as buried is 0.01 Å^2^, with a SASA probe radius of 1.2 Å.

### 4.5 Epitope prediction

Epitopes in the NTD region were predicted by the ElliPro Antibody Epitope Prediction server (Ponomarenko et al. 2008). NTDs are shown as predicted linear epitopes when using PDB accession codes 6VXX (Wrapp et al. 2020) and 6VSB (Walls et al. 2020), (structural coordinates corresponding to the entire S protein), along with a minimum score of 0.9, i.e. a highly strict criterion.

## Supplementary Material

veab069_Supp
