# Supplementary material for: The ongoing evolution of variants of concern and interest of SARS-CoV-2 in Brazil revealed by convergent indels in the amino (N)-terminal domain of the spike protein
Source: Virus Evol. 2021 Aug 14;7(2):veab069. doi: 10.1093/ve/veab069 (PMC8438916; doi:10.1093/ve/veab069)
Supplement: veab069_Supp [file veab069_supp.zip › Appendix Table 1.docx]

**Appendix Table 1. SARS-CoV-2 Brazilian variants with indels at NTD of the Spike protein.**

| **Sample(s)** | **Lineage** | **NTD Indel** | **RBD** | **GISAID ID** | **Cluster** |
| --- | --- | --- | --- | --- | --- |
| MG-FIOCRUZ-8180/2021  MA-FIOCRUZ-11517/2021 | P.2 | 𝚫144 | E484K | EPI_ISL_1219137  EPI_ISL_1465185 | -  - |
| SP-IB_100537/2021 | P.2 | 𝚫141-144 | E484K | EPI_ISL_1966552 | - |
| SC-FIOCRUZ-13109/2021  SC-FIOCRUZ-13111/2021  SC-FIOCRUZ-13113/2021  SC-FIOCRUZ-13114/2021  SC-FIOCRUZ-19105/2021  SC-FIOCRUZ-19128/2021  SP-2075/2021  SP-2084/2021  SP-2088/2021  SP-BT19579/2021  SP-BTSC-7/2021  SP-IB_100268/2021  SP-IB_100423/2021  SP-IB_100800/2021  SP-SJRP129800/2021  SP-IB_101267/2021 | P.1 | 𝚫69-70 | K417T E484K N501Y | EPI_ISL_1533994  EPI_ISL_1533992  EPI_ISL_1533996  EPI_ISL_1533993  EPI_ISL_2157547  EPI_ISL_2157548  EPI_ISL_1498917  EPI_ISL_1509639  EPI_ISL_1509720  EPI_ISL_1734844  EPI_ISL_2223449  EPI_ISL_1966297  EPI_ISL_1966444  EPI_ISL_1966787  EPI_ISL_2008924  EPI_ISL_2170977 | NC  NC  NC  NC  V  V  II  II  II  NC  NC  III  III  III  -  III |
| BA53/2021*  BA54/2021*  BA55/2021*  BA-FIOCRUZ-7029/2021*  AM-FIOCRUZ-21140861HC*  AM-IEC-177911/2021*  RJ-FIOCRUZ-14135/2021  RJ-LNN00339/2021  SP-833689/2021  SP-IB_102491/2021  SP-IB_100090/2021  RJ-LNN00793/2021  RJ-LNN01072/2021 | P.1    P.1 | 𝚫144    𝚫143-144 | K417T E484K N501Y    K417T E484K N501Y | EPI_ISL_1067729  EPI_ISL_1067733  EPI_ISL_1067734  EPI_ISL_1219136  EPI_ISL_1533609  EPI_ISL_1261694  EPI_ISL_2443587  EPI_ISL_1858688  EPI_ISL_1795391  EPI_ISL_2345528  EPI_ISL_1966135  EPI_ISL_2101733  EPI_ISL_2101734 | I  I  I  I  I  I  NC  NC  IV  IV  NC  II  NC |
| AL-FIOCRUZ-4795/2021*  PR-FIOCRUZ-5273/2021**  SP-IB_100743/2021 | P.1 | 𝚫141-144 | K417T E484K N501Y | EPI_ISL_1219134  EPI_ISL_1219133  EPI_ISL_1966737 | NC  NC  NC |
| AL-FIOCRUZ-4786/2021*  RS-FIOCRUZ-14243/2021 | P.1 | 𝚫189-190 | K417T E484K N501Y | EPI_ISL_1219135  EPI_ISL_1534013 | I  I |
| SE-FIOCRUZ-10220/2021    AM-FIOCRUZ-20897269OP*  AM-FIOCRUZ-20897281WS*  AM-FIOCRUZ-21840593CL*  PR-FIOCRUZ-5241/2021  SC-FUNED-16-786/2021  SP-IB_100668/2021 | P.1    P.1 | 𝚫242-244    ins214ANRN | K417T E484K N501Y  K417T E484K N501Y | EPI_ISL_1534004    EPI_ISL_1261123  EPI_ISL_1068256  EPI_ISL_1219132  EPI_ISL_1261122  EPI_ISL_2293000  EPI_ISL_1966669 | NC    P.1-like-I  P.1-like-I  P.1-like-I  P.1-like-I  P.1-like-I  P.1-like-I |
| MA-FIOCRUZ-6871/2021*** | N.10 | 𝚫141-144  𝚫211  𝚫256-258 | V445A  E484K | EPI_ISL_1181371 |  |

*Patient from Amazonas state or traveller returning from Amazonas state. ** Patient from Rondônia. *** Sequence representative of lineage N.10. Sequencing depth plots of the samples bearing indels are available in Supplementary Figure S1.
