# Supplementary material for: The ongoing evolution of variants of concern and interest of SARS-CoV-2 in Brazil revealed by convergent indels in the amino (N)-terminal domain of the spike protein
Source: Virus Evol. 2021 Aug 14;7(2):veab069. doi: 10.1093/ve/veab069 (PMC8438916; doi:10.1093/ve/veab069)
Supplement: veab069_Supp [file veab069_supp.zip › Appendix Table 2.docx]

**Appendix Table 2.** B.1.1.28, P.1 and P.2 genomes harboring NTD indels detected outside Brazil. Data retrieved from GISAID up to 31th May 2021.

| **Lineage** | **Countries** | **NTD Indel** | **N** | **Earliest Sequence** | **Last sequence** |
| --- | --- | --- | --- | --- | --- |
| B.1.1.28 | USA | Δ69-70 | 2 | 20th Apr 2021 | 24th Apr 2021 |
|  | Italy  Paraguay  USA | Δ144 | 4 | 21th Feb 2021 | 24th Apr 2021 |
|  | Turkey  USA | Δ242-244 | 2 | 10th Mar 2021 | 07th Apr 2021 |
| P.2 | Netherlands  USA | Δ69-70 | 2 | 13th Jan 2021 | 05th Mar 2021 |
|  | Paraguay  Portugal  USA | Δ144 | 6 | 21th Feb 2021 | 09th May 2021 |
|  | Ireland  USA | Δ143-144 | 2 | 20th Feb 2021 | 24th Feb 2021 |
|  | Argentina  USA | Δ141-144 | 2 | 27th Feb 2021 | 30th Mar 2021 |
| P.1 | Aruba  Austria  Spain  USA | Δ69-70 | 5 | 17th Jan 2021 | 24th May 2021 |
|  | Spain  USA | Δ144 | 2 | 05th Feb 2021 | 03th May 2021 |
|  | USA | Δ143-144 | 3 | 16th Apr 2021 | 30th Apr 2021 |
|  | USA | Δ141-143 | 62 | 11th Mar 2021 | 13th May 2021 |
|  | USA | Δ141-144 | 2 | 08th Apr 2021 | 13th Apr 2021 |
|  | France  USA | Δ139-144 | 6 | 02th Mar 2021 | 26th Apr 2021 |
|  | USA | Δ242-244 | 1 | 23th Mar 2021 | - |
| P.1  (P.1-like-I) | Guyana | ins214ANRN | 1 | 18th Jan 2021 | - |
