# Supplementary material for: The ongoing evolution of variants of concern and interest of SARS-CoV-2 in Brazil revealed by convergent indels in the amino (N)-terminal domain of the spike protein
Source: Virus Evol. 2021 Aug 14;7(2):veab069. doi: 10.1093/ve/veab069 (PMC8438916; doi:10.1093/ve/veab069)
Supplement: veab069_Supp [file veab069_supp.zip › Appendix Table 3.docx]

**Appendix Table 3.** Defining mutations of SARS-CoV-2 lineage N.10.

| **Genomic region (protein)** | **Nucleotide** | **Amino acid** |
| --- | --- | --- |
| ORF1ab (NSP3) | C5184T | P822L |
| ORF1ab (NSP5) | C10376T | P108S |
|  | C10478T | P132S |
| ORF1ab (NSP6) | T11418C | V149A |
| Spike (S) | C21588T | P9L |
|  | ∆21984 - 21996 | ∆141-144 |
|  | A22190G | I210V |
|  | ∆22193 - 22195 | ∆211 |
|  | T22196A | L212I |
|  | ∆22327 - 22336 | ∆256-258 |
|  | T22896C | V445A |
|  | G23012A | E484K |
| ORF7b (NSP7b) | ∆27794 - 27798 | frame-shifted and truncated |
| N | A28482G | Q70R |
