# Supplementary material for: The ongoing evolution of variants of concern and interest of SARS-CoV-2 in Brazil revealed by convergent indels in the amino (N)-terminal domain of the spike protein
Source: Virus Evol. 2021 Aug 14;7(2):veab069. doi: 10.1093/ve/veab069 (PMC8438916; doi:10.1093/ve/veab069)
Supplement: veab069_Supp [file veab069_supp.zip › Appendix Table 4.docx]

**Appendix Table 4.** Number of genomes harboring NTD indels in Brazil and the World. Data retrieved from GISAID up to 31th May 2021.

|  |  | **69-70 del** | **141-144del** | **143-144del** | **144del** | **189-190del** | **211del** | **ins214ANRN** | **242-244del** | **256-258del** |
| --- | --- | --- | --- | --- | --- | --- | --- | --- | --- | --- |
| **Brazil** | **N** | 307 | 29 | 30 | 340 | 2 | 23 | 6 | 4 | 24 |
|  | **%** | 2.41 | 0.23 | 0.24 | 2.66 | 0.02 | 0.18 | 0.05 | 0.03 | 0.19 |
|  | **First** | 2020-05-18 | 2020-12-29 | 2020-12-29 | 2020-06-19 | 2021-01-27 | 2020-12-29 | 2020-12-23 | 2021-02-03 | 2020-12-29 |
|  | **Last** | 2021-05-11 | 2021-04-19 | 2021-04-19 | 2021-05-11 | 2021-03-15 | 2021-04-19 | 2021-04-05 | 2021-03-10 | 2021-04-26 |
|  | **Lineages** | B.1.1, B.1.1.28, B.1.1.7, B.1.525, P.1 | B.1.1.33, N.10, P.1, P.1.2, P.2 | B.1.1.33, B.1.1.7, N.10, P.1, P.1.2, P.2 | B.1.1, B.1.1.28, B.1.1.33, B.1.1.7, B.1.525, C.29, N.10, P.1, P.1.2, P.2 | P.1 | N.10 | P.1 (P.1-like-I) | P.1, B.1.351 | N.10 |
| **World** | **N** | 773405 | 731 | 818 | 761570 | 14 | 156 | 7 | 18934 | 38 |
|  | **%** | 44.2 | 0.04 | 0.05 | 43.52 | 8.00E-04 | 0.01 | 4.00E-04 | 1.08 | 0 |
|  | **First** | 2020-01-05 | 2020-02-25 | 2020-02-25 | 2020-01-27 | 2020-12-28 | 2020-04-02 | 2020-12-23 | 2020-03-26 | 2020-04-24 |
|  | **Last** | 2021-05-31 | 2021-05-25 | 2021-05-25 | 2021-05-31 | 2021-05-11 | 2021-05-16 | 2021-04-05 | 2021-05-26 | 2021-05-12 |
|  | **Lineages (N)** | 219 | 130 | 142 | 392 | 5* | 21 | 1^#^ | 87 | 6^†^ |

*B.1, B.1.1.7, B.1.177, B.1.617.2, P.1

^#^ P.1 (P.1-like-I)

^†^ B.1, B.1.1, B.1.1.7, B.1.595, C.37, N.10
