# Supplementary material for: The ongoing evolution of variants of concern and interest of SARS-CoV-2 in Brazil revealed by convergent indels in the amino (N)-terminal domain of the spike protein
Source: Virus Evol. 2021 Aug 14;7(2):veab069. doi: 10.1093/ve/veab069 (PMC8438916; doi:10.1093/ve/veab069)
Supplement: veab069_Supp [file veab069_supp.zip › Appendix Table 5.docx]

**Appendix Table 5.** SARS-CoV-2 sequences available at EpiCoV database in the GISAID that displayed an insert motif in positions 214-216 of the Spike protein.

| **Lineage** | **Number** | **Motif** | **First** | **Last** |
| --- | --- | --- | --- | --- |
| A.2.5 | 656 | AAG | 12 Apr 2020 | 20 May 2021 |
| B | 1 | AKKN | 05 Mar 2020 | - |
| B | 3 | KRI | 28 Dec 2020 | 15 Mar 2021 |
| B.1 | 55 | AAG/TDR/QAS/DRS | 18 Jan 2021 | 26 Mar 2021 |
| B.1.1.7 | 8 | AAG/APR/KFH /KAFKQ/ER | 12 Feb 2021 | 13 May 2021 |
| B.1.1.519 | 1 | KGE | 27 Apr 2021 | - |
| B.1.143 | 1 | TDR | 17 Feb 2021 | - |
| B.1.177 | 1 | KLGP | 13 Nov 2020 | - |
| B.1.2 | 5 | ADL/NFG | 31 Jan 2021 | Mar 2021 |
| B.1.214 | 875 | TDR/QID | 22 Nov 2020 | 26 May 2021 |
| B.1.221 | 4 | QAS | 13 Mar 2021 | 24 Mar 2021 |
| B.1.258 | 2 | TDR | 18 Jan 2021 | - |
| B.1.427/429 | 2 | AAG/AQER | 15 Jan 2021 | 18 Mar 2021 |
| B.1.459 | 1 | TDR | 30 Mar 2021 | - |
| B.1.499 | 1 | AAG | 01 Mar 2021 | - |
| B.1.526 | 2 | TDR | 27 Mar 2021 | 18 Apr 2021 |
| C.37 | 1 | TDR | 09 Apr 2021 | - |
