# Supplementary material for: The ongoing evolution of variants of concern and interest of SARS-CoV-2 in Brazil revealed by convergent indels in the amino (N)-terminal domain of the spike protein
Source: Virus Evol. 2021 Aug 14;7(2):veab069. doi: 10.1093/ve/veab069 (PMC8438916; doi:10.1093/ve/veab069)
Supplement: veab069_Supp [file veab069_supp.zip › Appendix Table 7.pdf]

We gratefully acknowledge the following Authors from the Originating laboratories responsible for obtaining the specimens, as well as the Submitting laboratories where the genome data were generated and shared via GISAID, on which this research is based.

All Submitters of data may be contacted directly via www.gisaid.org

Authors are sorted alphabetically.

Accession ID	Originating Laboratory	Submitting Laboratory	Authors
EPI_ISL_402131	Wuhan Institute of Virology, Chinese Academy of Sciences	Wuhan Institute of Virology, Chinese Academy of Sciences	Yan Zhu, Ping Yu, Bei Li, Ben Hu, Hao-Rui Si, Xing-Lou Yang, Peng Zhou, Zheng-Li Shi
EPI_ISL_410539, EPI_ISL_410541	Beijing Institute of Microbiology and Epidemiology	Beijing Institute of Microbiology and Epidemiology	Wu-Chun Cao; Tommy Tsan-Yuk Lam; Na Jia; Ya-Wei Zhang; Jia-Fu Jiang; Bao-Gui Jiang
EPI_ISL_412977	Shandong First Medical University & Shandong Academy of Medical Sciences	Institute of Microbiology, Chinese Academy of Sciences	Weifeng Shi, Tao Hu, Hong Zhou, Juan Li, Xing Chen, Alice Catherine Hughes, Yuhai Bi
EPI_ISL_471469, EPI_ISL_471470	South China Agricultural University	South China Agricultural University	Yongyi Shen, Wu Chen
EPI_ISL_852604, EPI_ISL_852605	Virology Unit, Institut Pasteur du Cambodge	G5 Evolutionary Genomics of RNA viruses, Virology Department, Institut Pasteur	Vibol Hul, Deborah Delaune, Erik A Karlsson, Ou Tey Putita, Alexandre Hassanin, Artem Baidaliuk, Fabiana Gámbaro, Vuong Tan Tu, Lucy Keatts, Jonna Mazet, Christine Johnson, Philippe Buchy, Philippe Dussart, Tracey Goldstein, Etienne Simon-Lorière, Veasna Duong
