# Supplementary material for: The ongoing evolution of variants of concern and interest of SARS-CoV-2 in Brazil revealed by convergent indels in the amino (N)-terminal domain of the spike protein
Source: Virus Evol. 2021 Aug 14;7(2):veab069. doi: 10.1093/ve/veab069 (PMC8438916; doi:10.1093/ve/veab069)
Supplement: veab069_Supp [file veab069_supp.zip › Supplementary Figure S1.pdf]

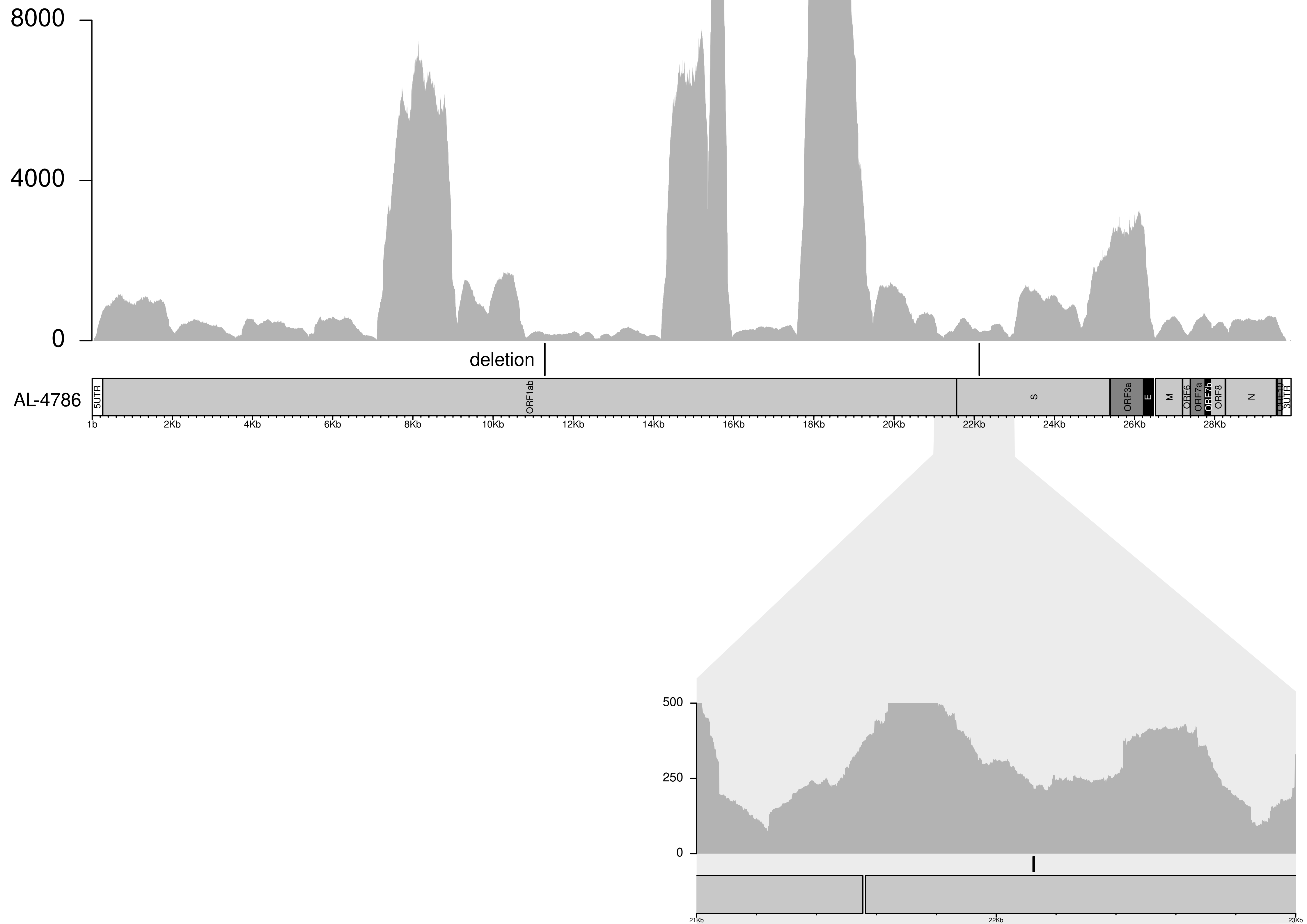
8000
4000
0
deletion
AL-4786
5UTR
ORF1ab
S
ORF3a
E
M
ORF6
ORF7a
ORF7b
ORF8
N
3UTR
1b
2Kb
4Kb
6Kb
8Kb
10Kb
12Kb
14Kb
16Kb
18Kb
20Kb
22Kb
24Kb
26Kb
28Kb
500
250
0
21Kb
22Kb
23Kb

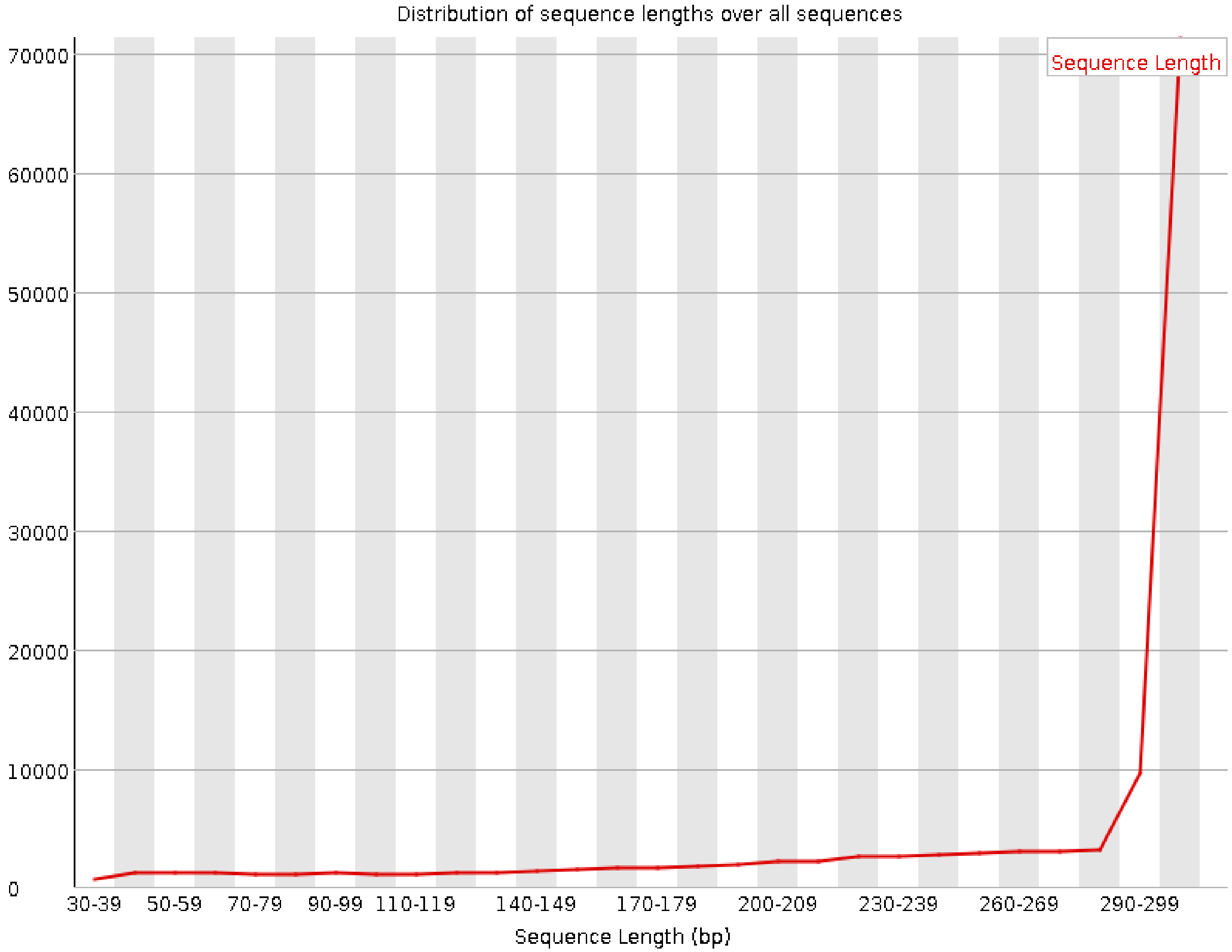
Distribution of sequence lengths over all sequences
Sequence Length
70000
60000
50000
40000
30000
20000
10000
0
30-39
50-59
70-79
90-99
110-119
140-149
170-179
200-209
230-239
260-269
290-299
Sequence Length (bp)

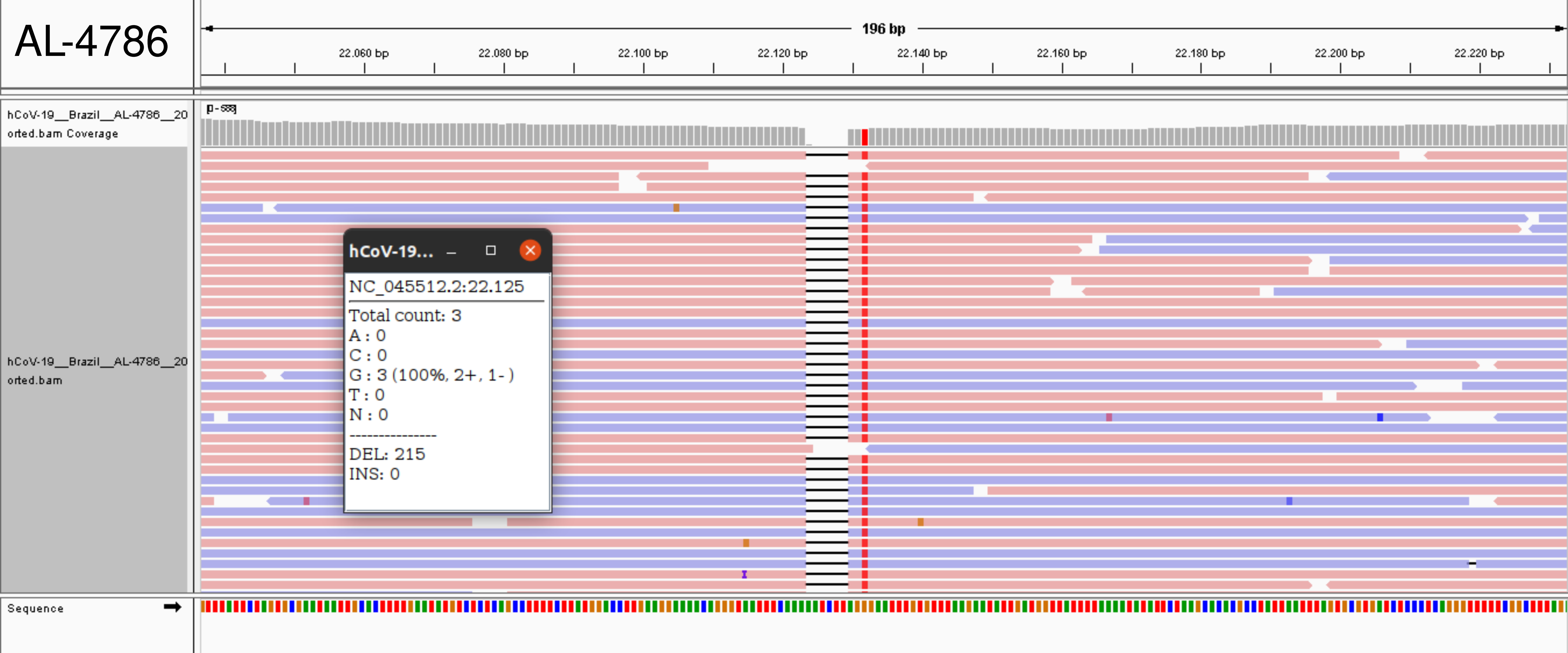
AL-4786
196 bp
22.060 bp
22.080 bp
22.100 bp
22.120 bp
22.140 bp
22.160 bp
22.180 bp
22.200 bp
22.220 bp
hCoV-19__Brazil__AL-4786__20
orted.bam Coverage
hCoV-19__Brazil__AL-4786__20
orted.bam
Sequence
hCoV-19...
NC_045512.2:22.125
Total count: 3
A : 0
C : 0
G : 3 (100%, 2+, 1-)
T : 0
N : 0
DEL: 215
INS: 0

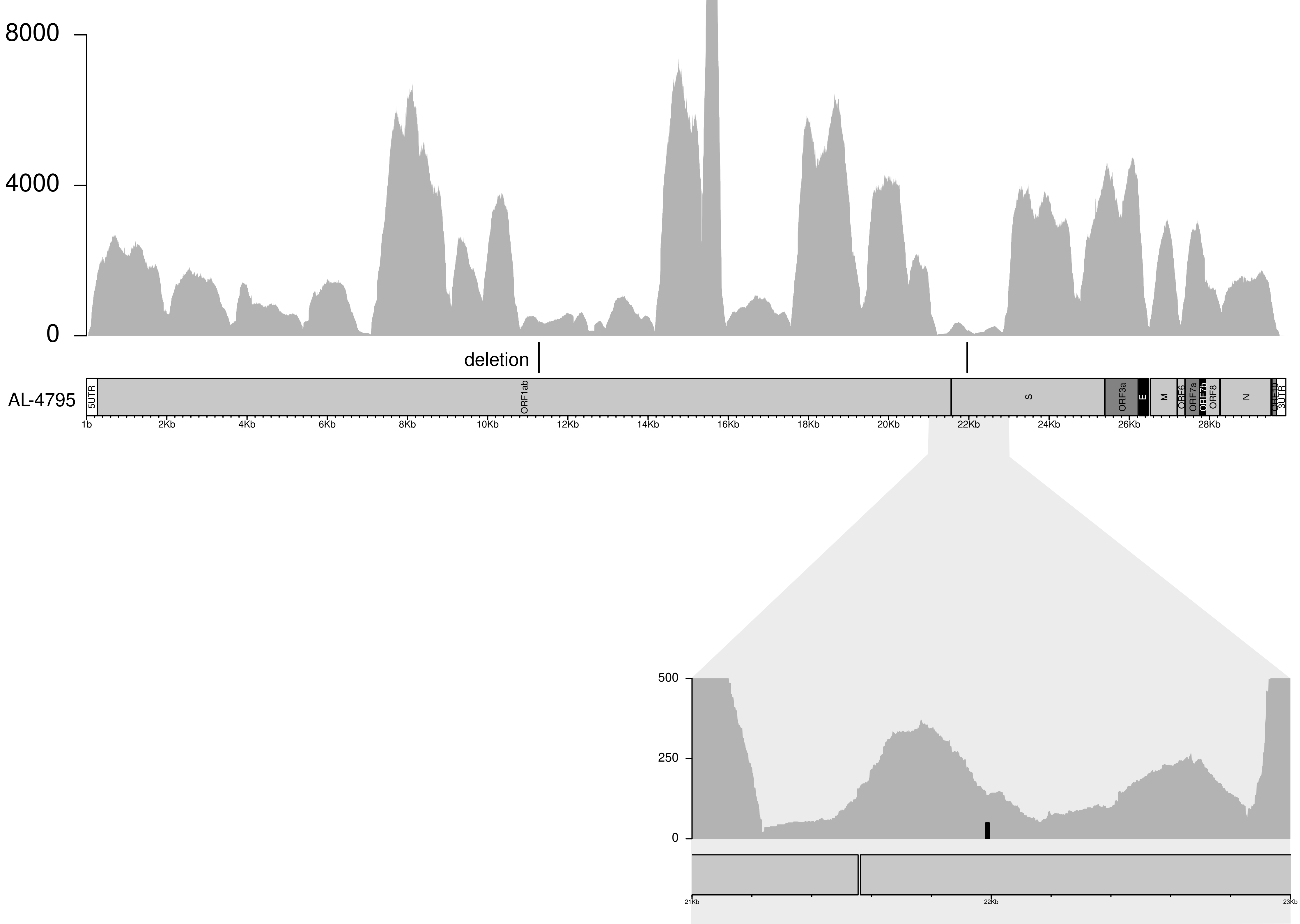
8000
4000
0
deletion
AL-4795
5UTR
ORF1ab
S
ORF3a
E
M
ORF6
ORF7a
ORF7b
ORF8
N
ORF10
3UTR
1b
2Kb
4Kb
6Kb
8Kb
10Kb
12Kb
14Kb
16Kb
18Kb
20Kb
22Kb
24Kb
26Kb
28Kb
500
250
0
21Kb
22Kb
23Kb

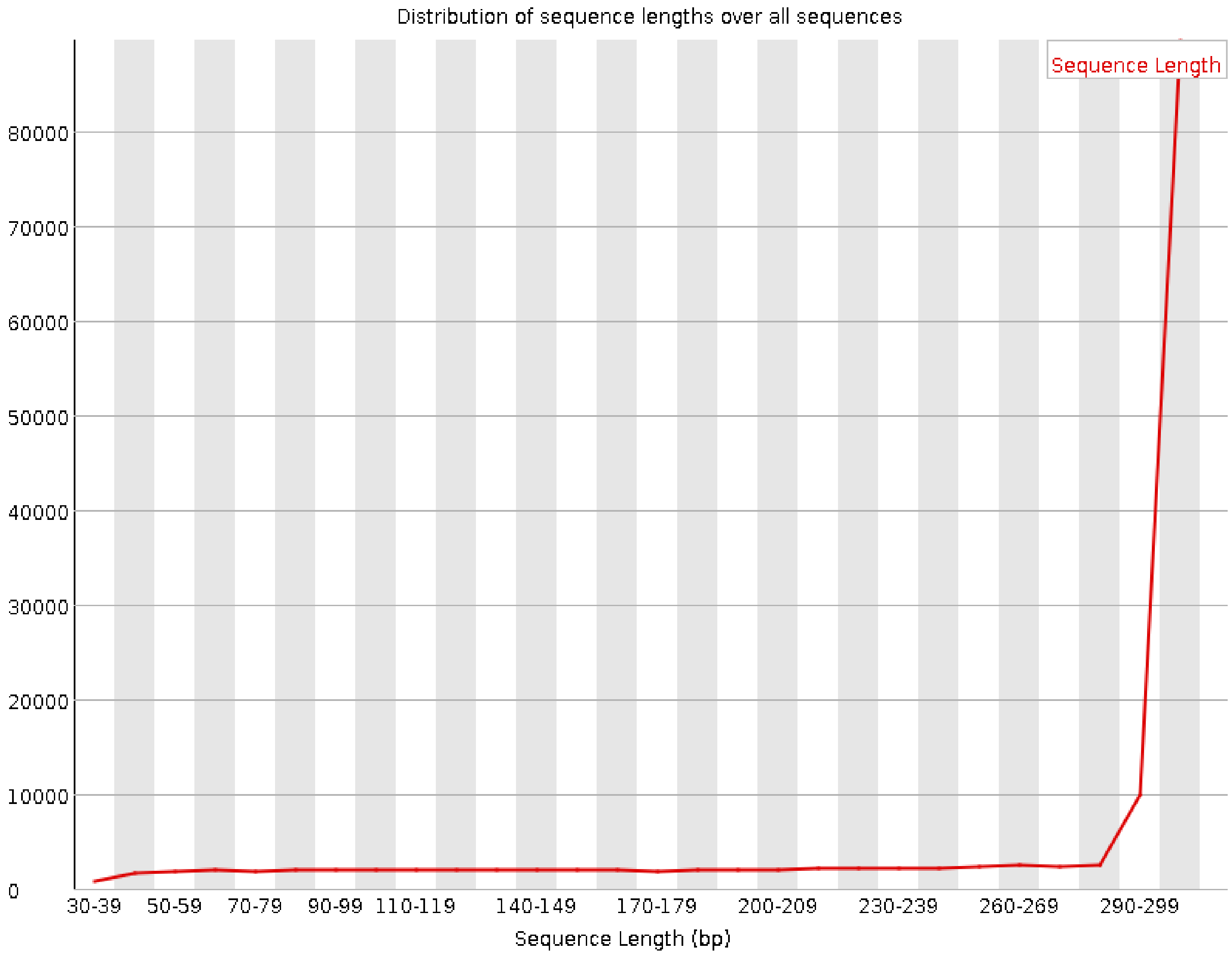
Distribution of sequence lengths over all sequences
Sequence Length
80000
70000
60000
50000
40000
30000
20000
10000
0
30-39
50-59
70-79
90-99
110-119
140-149
170-179
200-209
230-239
260-269
290-299
Sequence Length (bp)

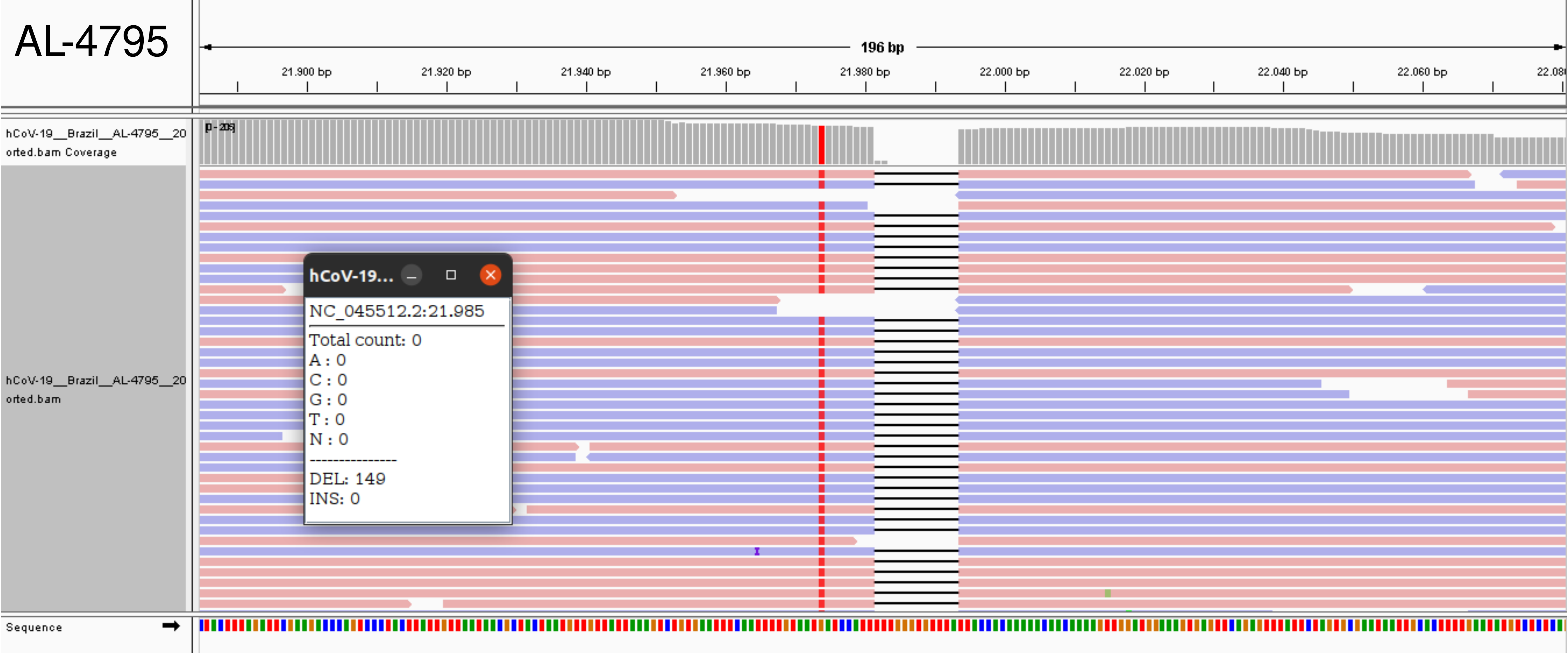
AL-4795
196 bp
21.900 bp
21.920 bp
21.940 bp
21.960 bp
21.980 bp
22.000 bp
22.020 bp
22.040 bp
22.060 bp
hCoV-19__Brazil__AL-4795__20
orted.bam Coverage
[0 - 208]
hCoV-19__Brazil__AL-4795__20
orted.bam
hCoV-19...
NC_045512.2:21.985
Total count: 0
A : 0
C : 0
G : 0
T : 0
N : 0

DEL: 149
INS: 0
Sequence

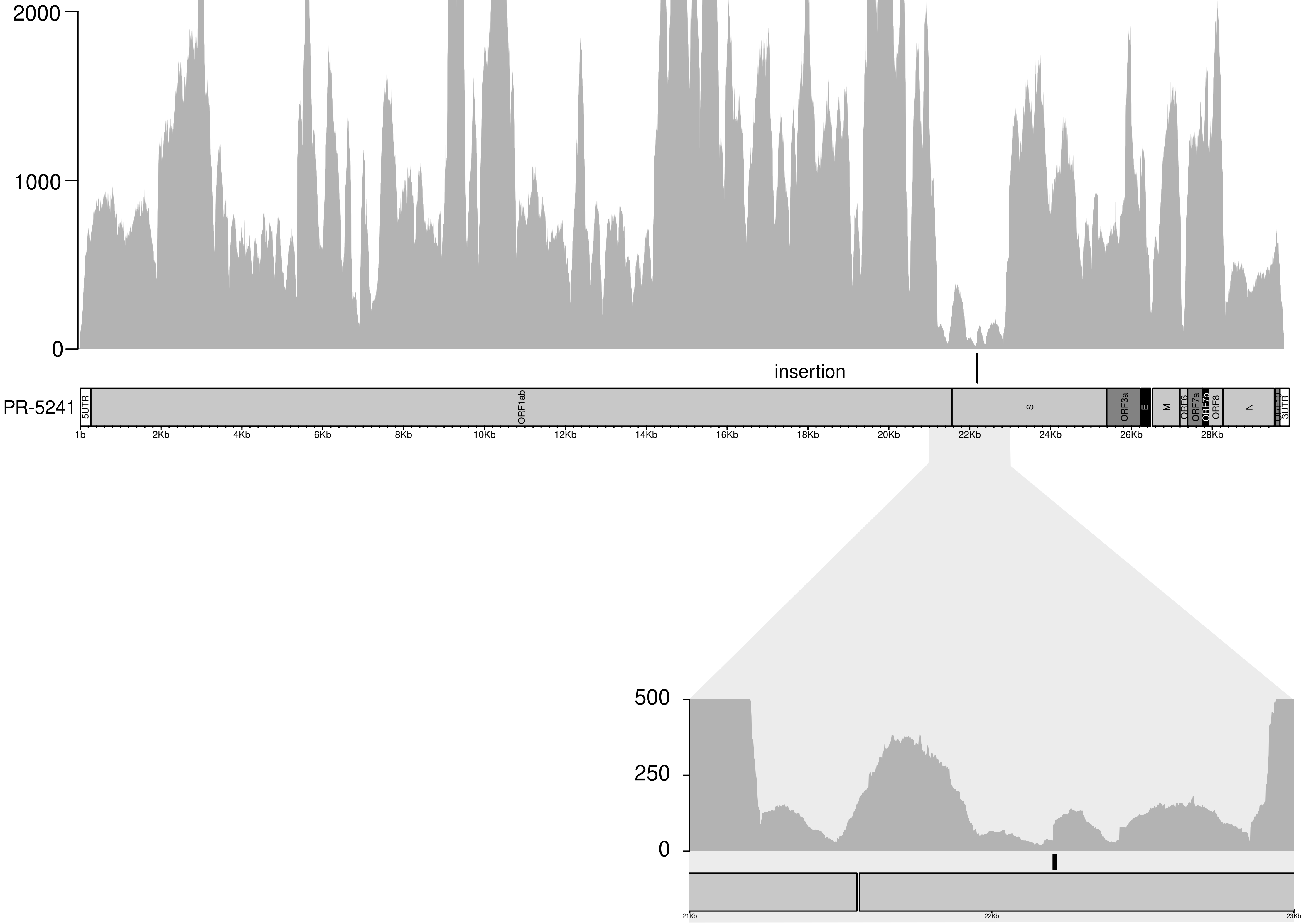
2000
1000
0
insertion
PR-5241
5UTR
ORF1ab
S
ORF3a
E
M
ORF6
ORF7a
ORF7b
ORF8
N
ORF10
3UTR
1b
2Kb
4Kb
6Kb
8Kb
10Kb
12Kb
14Kb
16Kb
18Kb
20Kb
22Kb
24Kb
26Kb
28Kb
500
250
0
21Kb
22Kb
23Kb

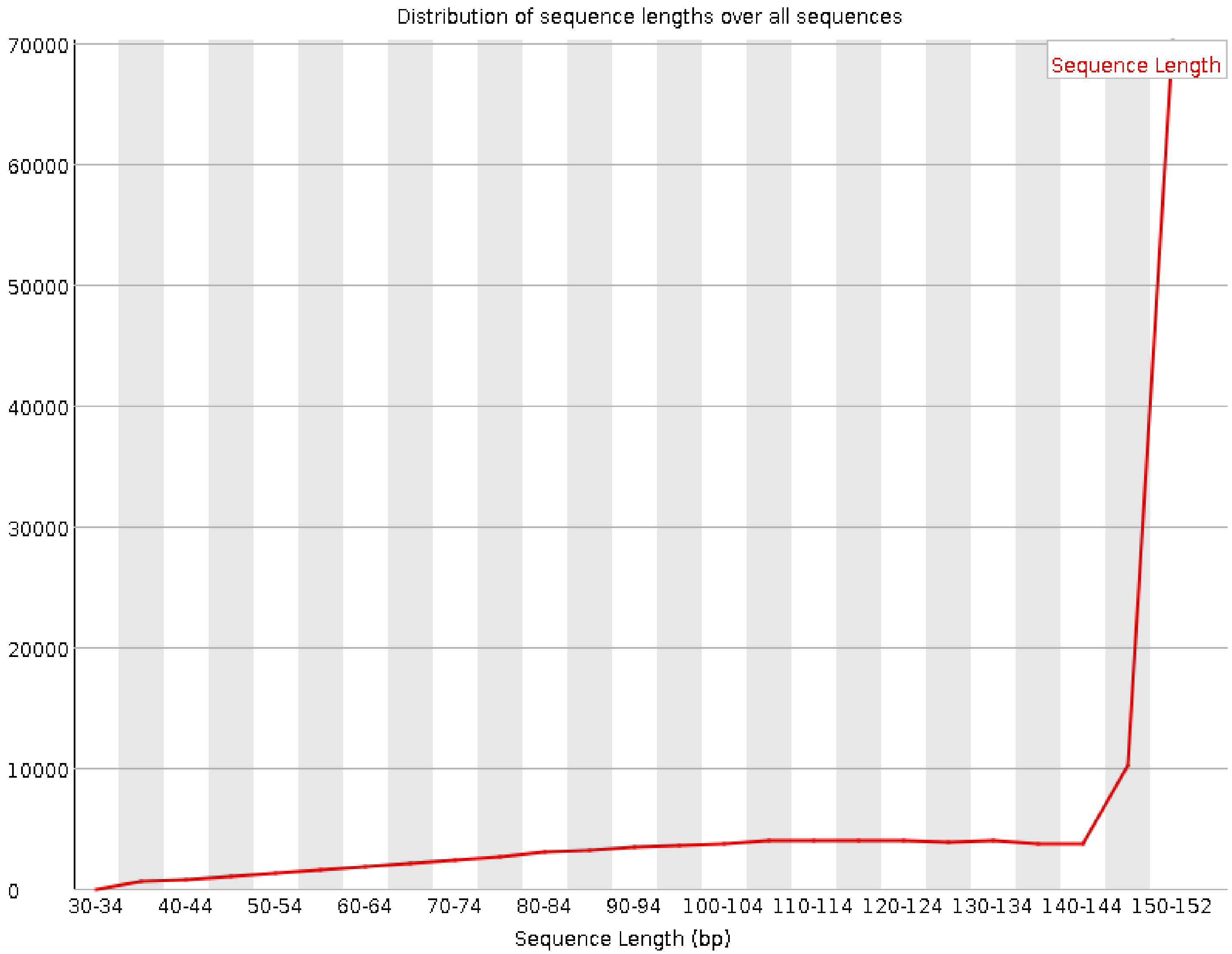
Distribution of sequence lengths over all sequences
Sequence Length
70000
60000
50000
40000
30000
20000
10000
0
30-34
40-44
50-54
60-64
70-74
80-84
90-94
100-104
110-114
120-124
130-134
140-144
150-152
Sequence Length (bp)

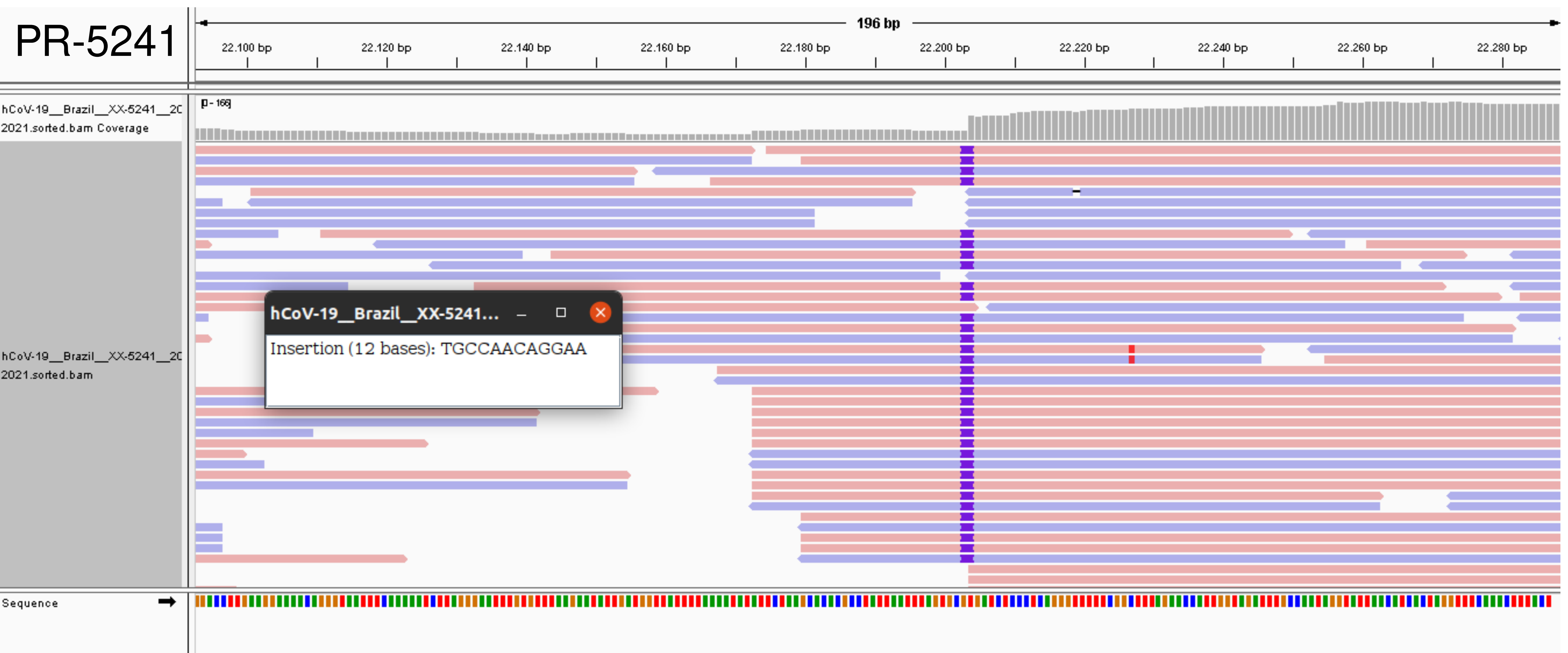

PR-5241
196 bp
22.100 bp
22.120 bp
22.140 bp
22.160 bp
22.180 bp
22.200 bp
22.220 bp
22.240 bp
22.260 bp
22.280 bp
[0 - 166]
hCoV-19__Brazil__XX-5241__20
2021.sorted.bam Coverage
hCoV-19__Brazil__XX-5241__20
2021.sorted.bam
hCoV-19__Brazil__XX-5241...
Insertion (12 bases): TGCCAACAGGAA
Sequence

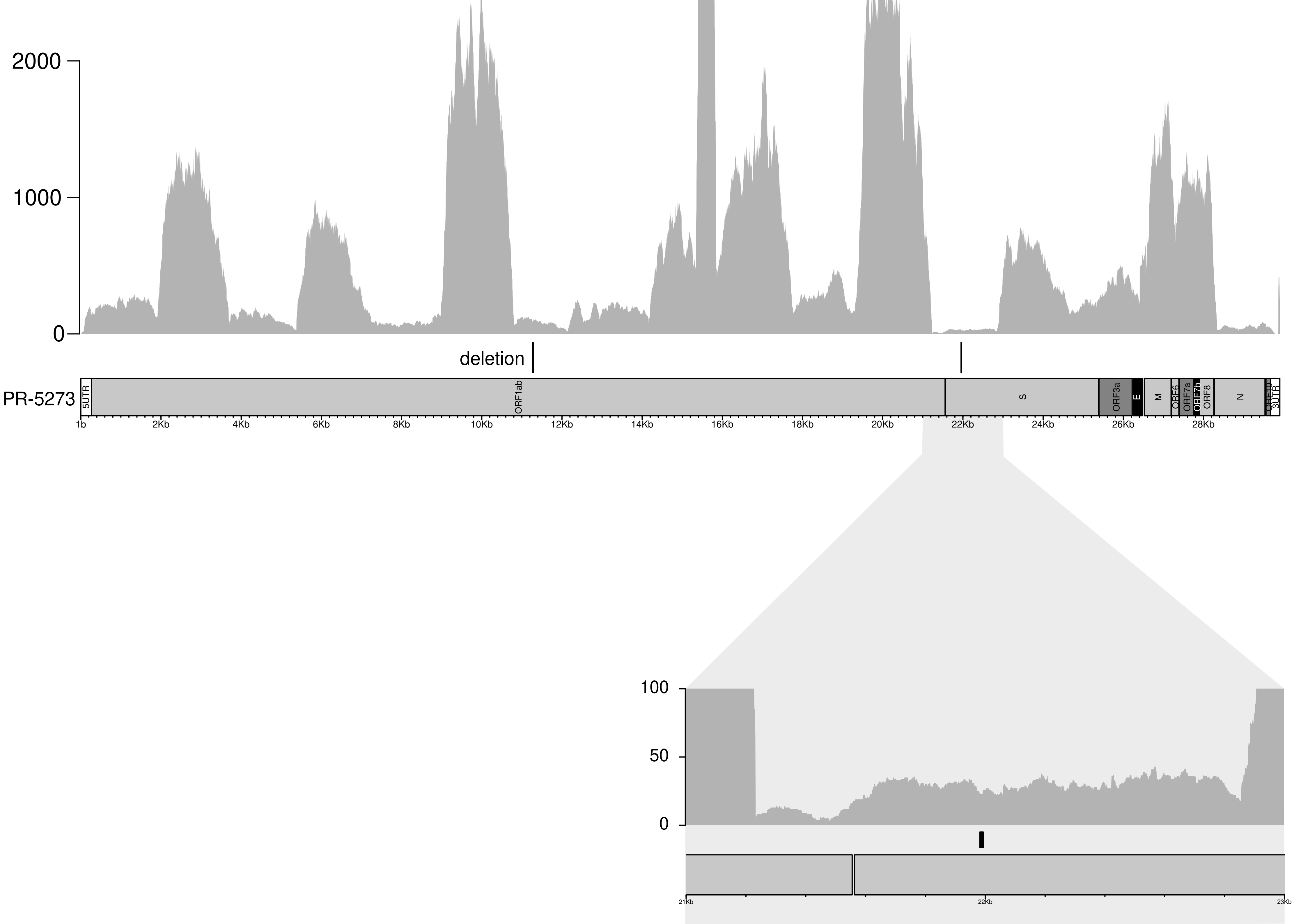

2000
1000
0
deletion
PR-5273
5UTR
ORF1ab
S
ORF3a
E
M
ORF6
ORF7a
ORF7b
ORF8
N
ORF10
3UTR
1b
2Kb
4Kb
6Kb
8Kb
10Kb
12Kb
14Kb
16Kb
18Kb
20Kb
22Kb
24Kb
26Kb
28Kb
100
50
0
21Kb
22Kb
23Kb

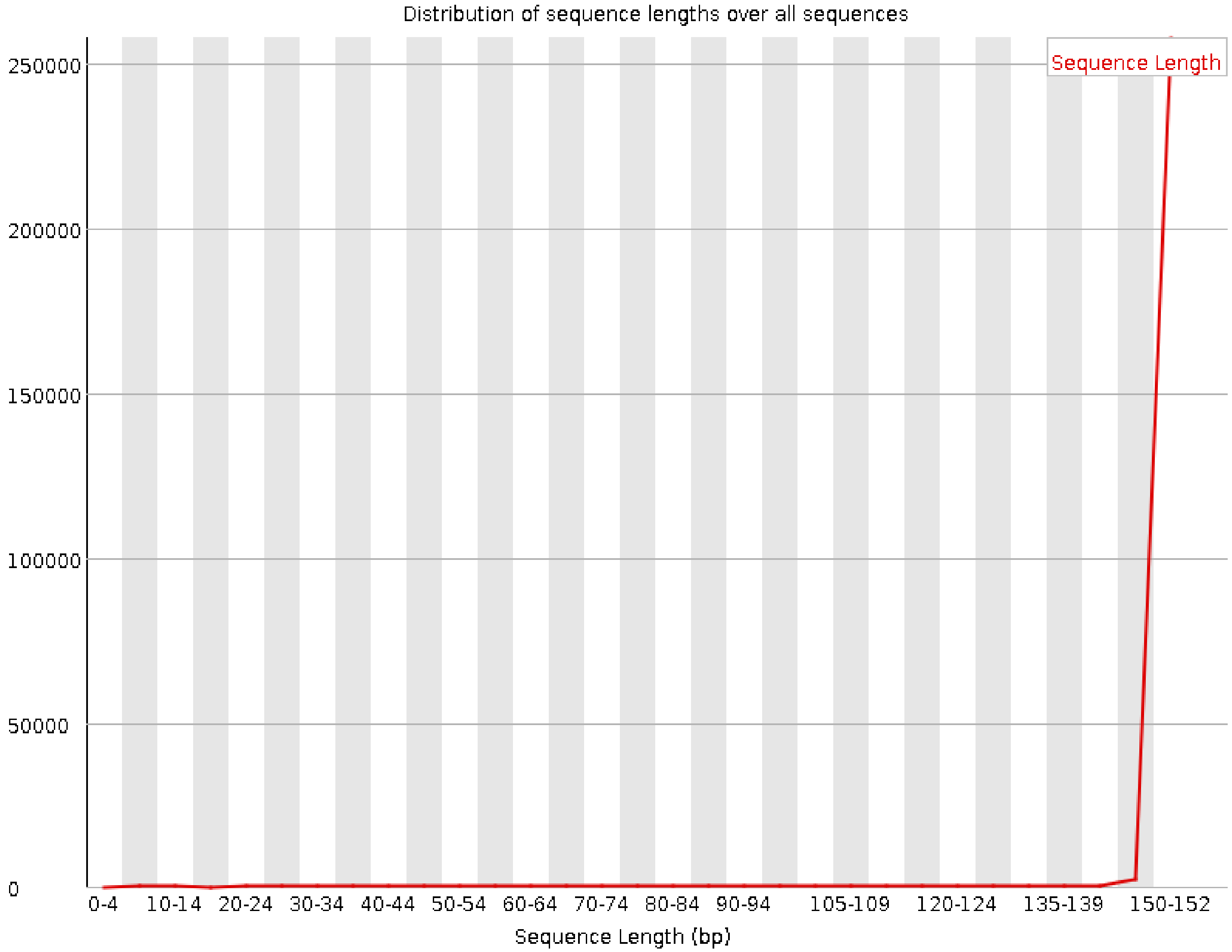
Distribution of sequence lengths over all sequences
Sequence Length
250000
200000
150000
100000
50000
0
0-4
10-14
20-24
30-34
40-44
50-54
60-64
70-74
80-84
90-94
105-109
120-124
135-139
150-152
Sequence Length (bp)

PR-5273

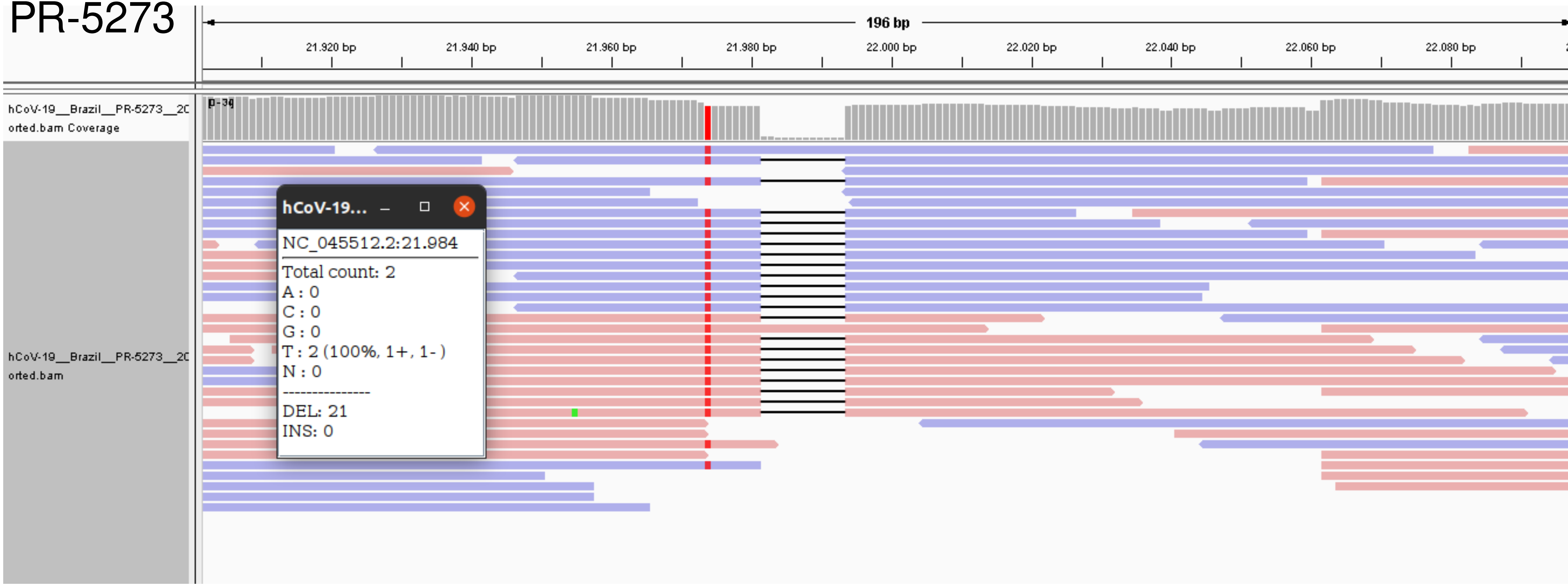
196 bp
21.920 bp
21.940 bp
21.960 bp
21.980 bp
22.000 bp
22.020 bp
22.040 bp
22.060 bp
22.080 bp
hCoV-19__Brazil__PR-5273__20
orted.bam Coverage
hCoV-19__Brazil__PR-5273__20
orted.bam
hCoV-19...
NC_045512.2:21.984
Total count: 2
A : 0
C : 0
G : 0
T : 2 (100%, 1+, 1-)
N : 0
DEL: 21
INS: 0

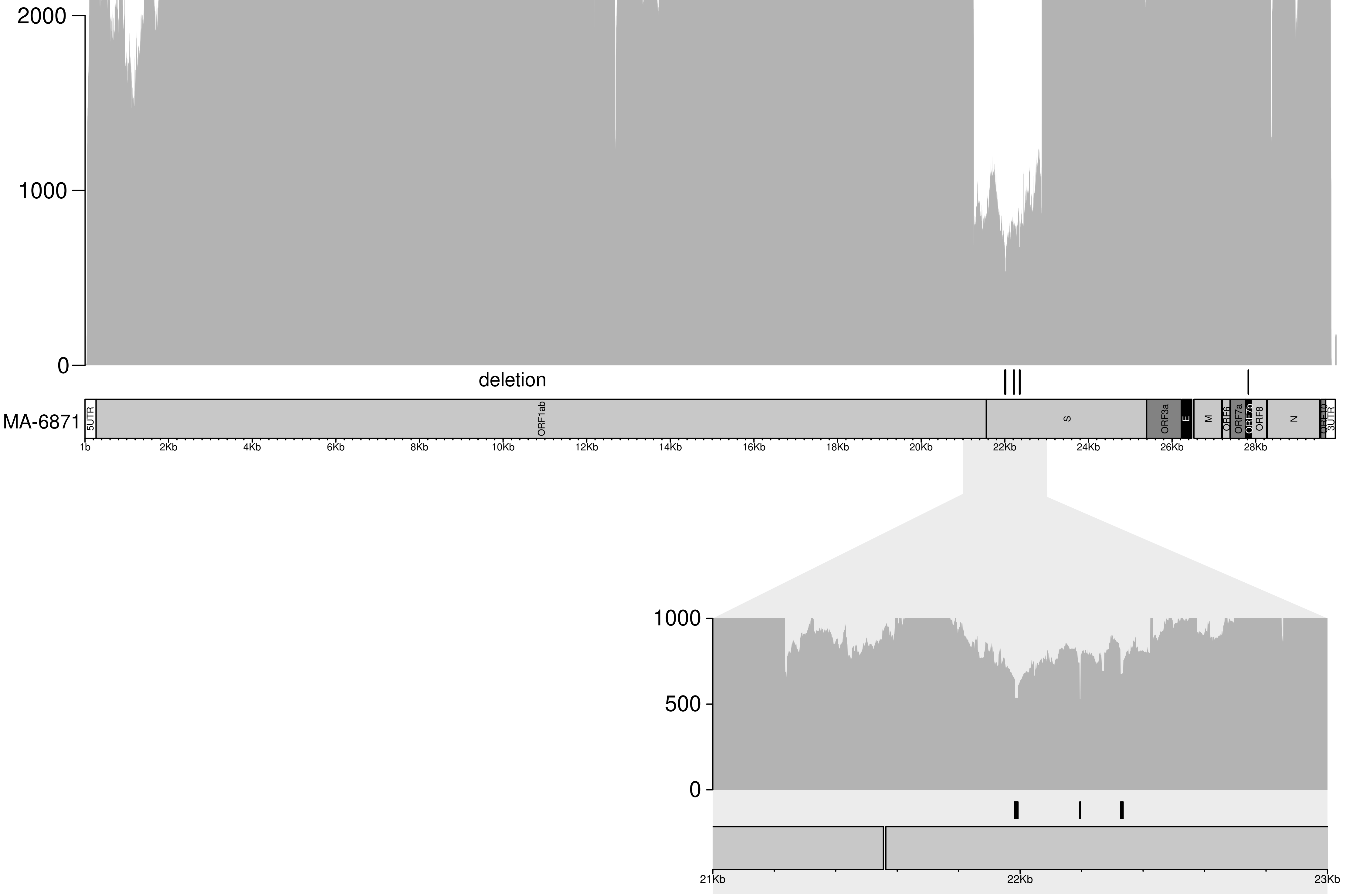

2000
1000
0
deletion
MA-6871
5UTR
ORF1ab
S
ORF3a
E
M
ORF6
ORF7a
ORF7b
ORF8
N
ORF10
3UTR
1b
2Kb
4Kb
6Kb
8Kb
10Kb
12Kb
14Kb
16Kb
18Kb
20Kb
22Kb
24Kb
26Kb
28Kb
1000
500
0
21Kb
22Kb
23Kb

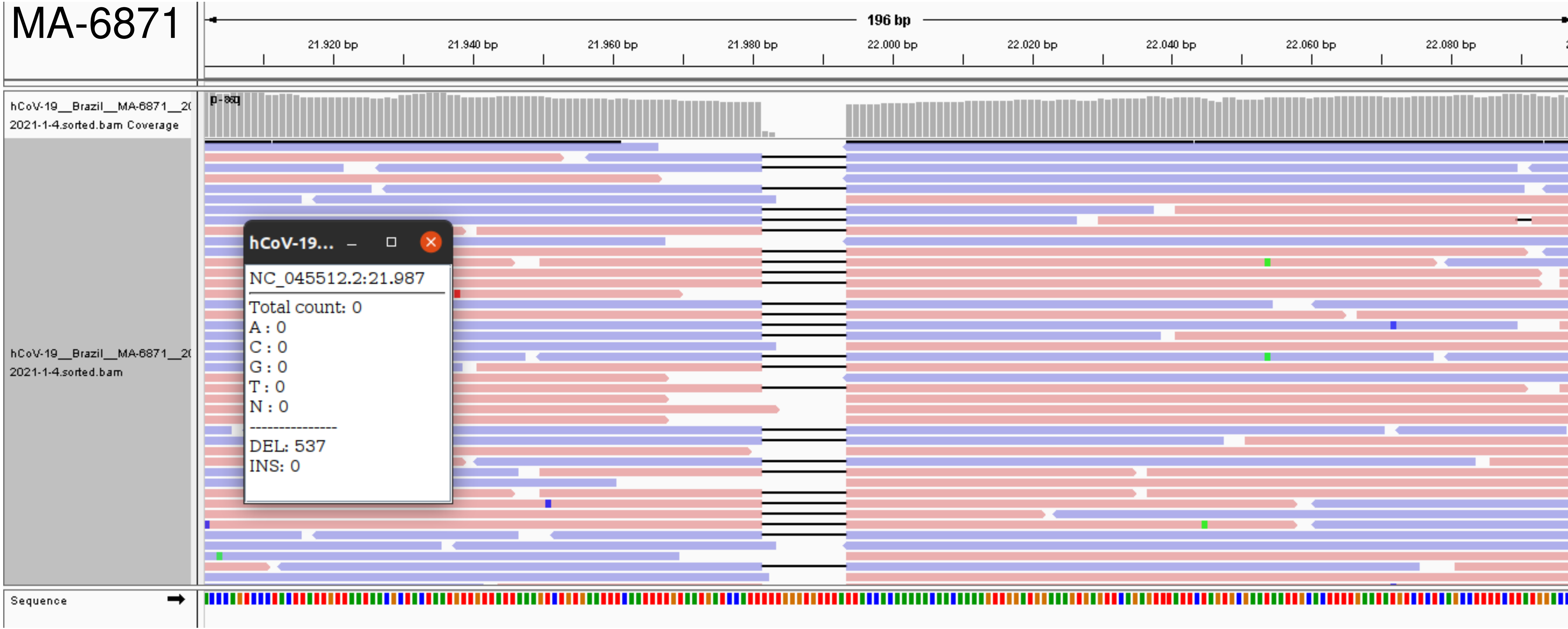
MA-6871
196 bp
21.920 bp
21.940 bp
21.960 bp
21.980 bp
22.000 bp
22.020 bp
22.040 bp
22.060 bp
22.080 bp
hCoV-19__Brazil__MA-6871__20
2021-1-4.sorted.bam Coverage
hCoV-19__Brazil__MA-6871__20
2021-1-4.sorted.bam
hCoV-19...
NC_045512.2:21.987
Total count: 0
A : 0
C : 0
G : 0
T : 0
N : 0
DEL: 537
INS: 0
Sequence

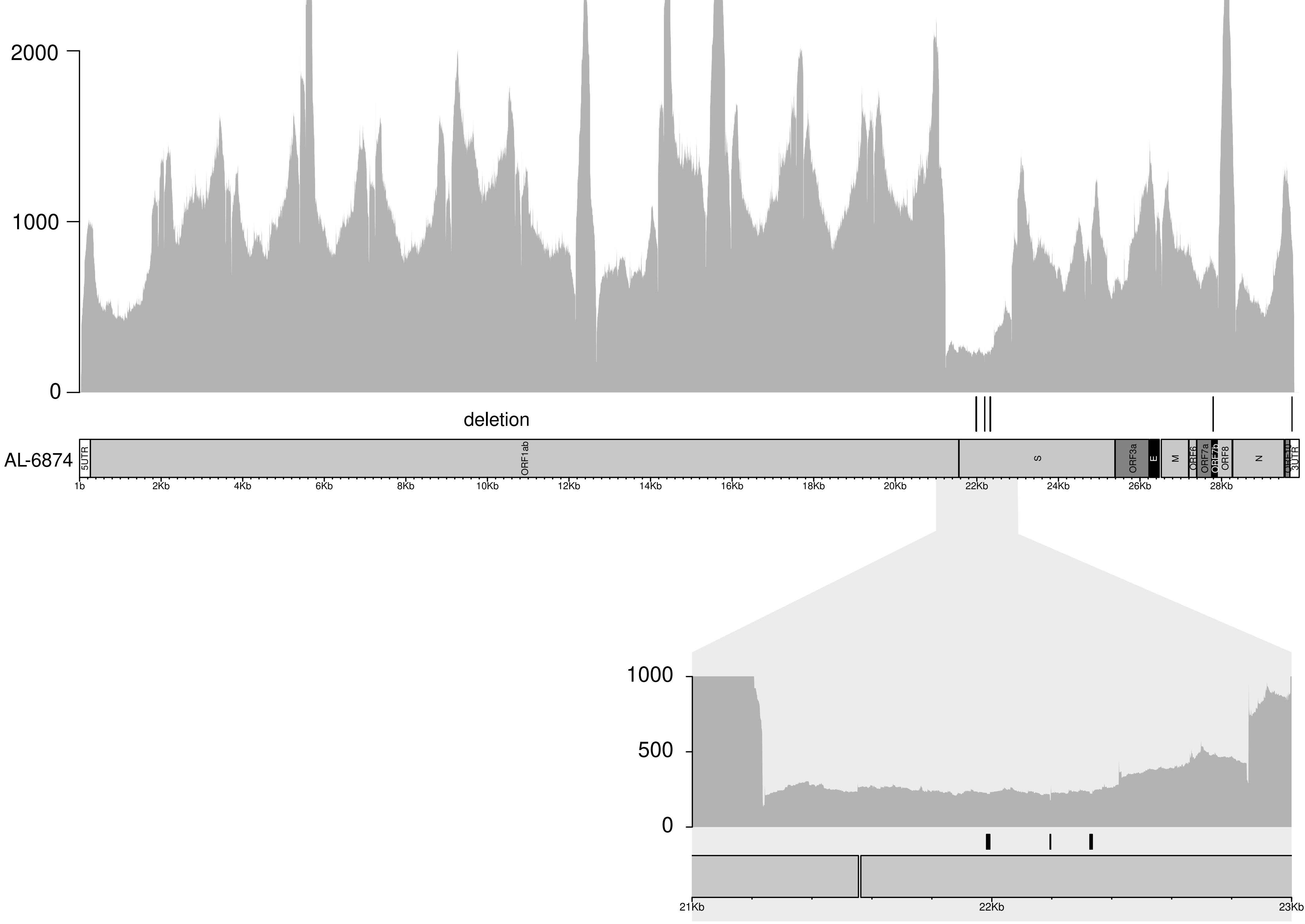
2000
1000
0
deletion
AL-6874
5UTR
ORF1ab
S
ORF3a
E
M
ORF6
ORF7a
ORF7b
ORF8
N
ORF10
3UTR
1b
2Kb
4Kb
6Kb
8Kb
10Kb
12Kb
14Kb
16Kb
18Kb
20Kb
22Kb
24Kb
26Kb
28Kb
1000
500
0
21Kb
22Kb
23Kb

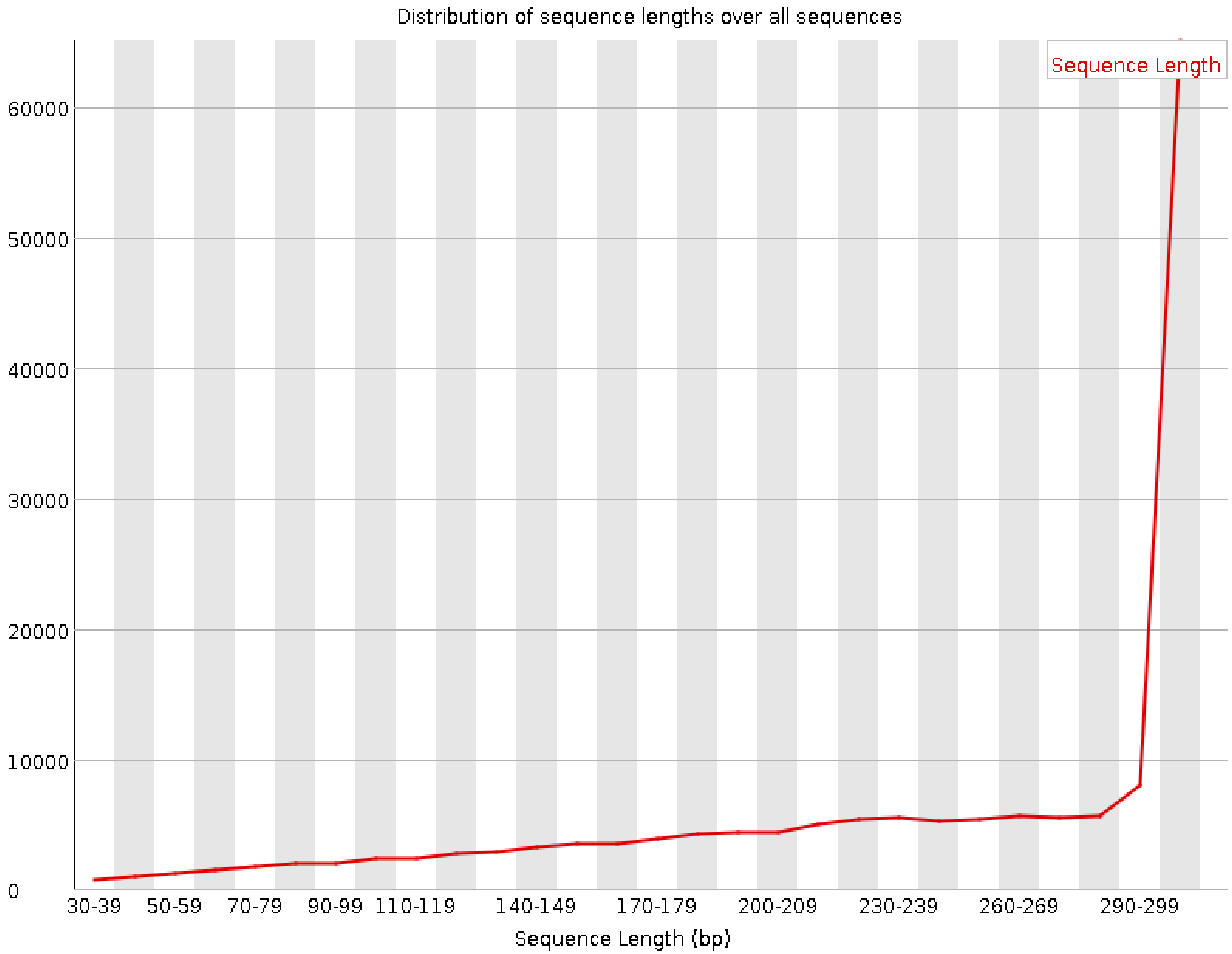
Distribution of sequence lengths over all sequences
Sequence Length
60000
50000
40000
30000
20000
10000
0
30-39
50-59
70-79
90-99
110-119
140-149
170-179
200-209
230-239
260-269
290-299
Sequence Length (bp)

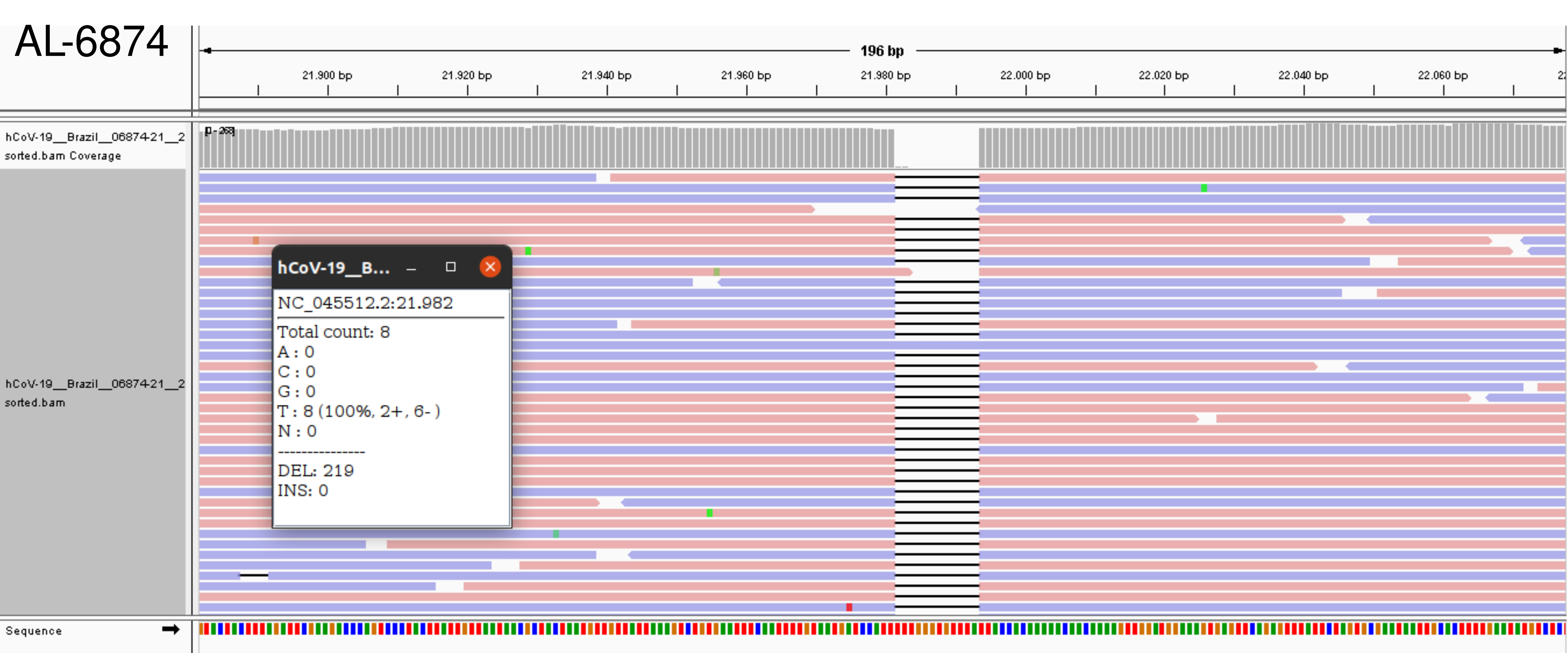
AL-6874
196 bp
21.900 bp
21.920 bp
21.940 bp
21.960 bp
21.980 bp
22.000 bp
22.020 bp
22.040 bp
22.060 bp
hCoV-19__Brazil__06874-21__2
sorted.bam Coverage
hCoV-19__Brazil__06874-21__2
sorted.bam
hCoV-19__B...
NC_045512.2:21.982
Total count: 8
A : 0
C : 0
G : 0
T : 8 (100%, 2+, 6-)
N : 0
DEL: 219
INS: 0
Sequence
