# Supplementary material for: The ongoing evolution of variants of concern and interest of SARS-CoV-2 in Brazil revealed by convergent indels in the amino (N)-terminal domain of the spike protein
Source: Virus Evol. 2021 Aug 14;7(2):veab069. doi: 10.1093/ve/veab069 (PMC8438916; doi:10.1093/ve/veab069)
Supplement: veab069_Supp [file veab069_supp.zip › Supplementary Figure S2.pdf]

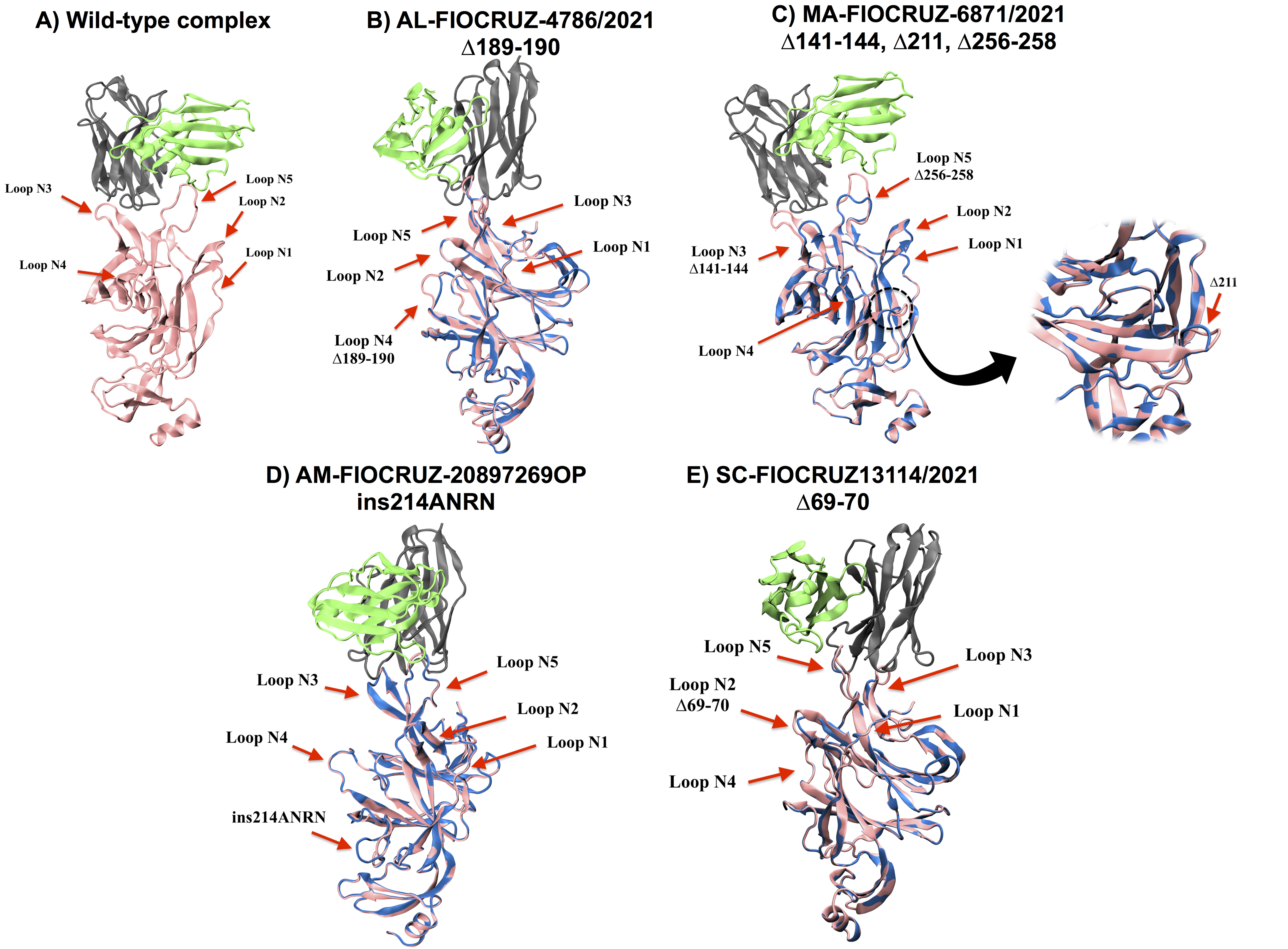
A) Wild-type complex
Loop N3
Loop N5
Loop N2
Loop N1
Loop N4
B) AL-FIOCRUZ-4786/2021
Δ189-190
Loop N3
Loop N5
Loop N1
Loop N2
Loop N4
Δ189-190
C) MA-FIOCRUZ-6871/2021
Δ141-144, Δ211, Δ256-258
Loop N5
Δ256-258
Loop N2
Loop N3
Δ141-144
Loop N1
Δ211
Loop N4
D) AM-FIOCRUZ-20897269OP
ins214ANRN
Loop N5
Loop N3
Loop N2
Loop N4
Loop N1
ins214ANRN
E) SC-FIOCRUZ13114/2021
Δ69-70
Loop N5
Loop N3
Loop N2
Δ69-70
Loop N1
Loop N4
